# Supplementary material for: Relationships between socioeconomic position and objectively measured sedentary behaviour in older adults in three prospective cohorts
Source: BMJ Open. 2017 Jun 15;7(6):e016436. doi: 10.1136/bmjopen-2017-016436 (PMC5541575; doi:10.1136/bmjopen-2017-016436)
Supplement: Supplementary Appendix 1 [file bmjopen-2017-016436supp001.pdf]

APPENDIX 1: ADDITIONAL DETAILS ON THE COLLECTION AND DERIVATION OF SEP MEASURES

The socioeconomic position (SEP) measures used in this study are as follows:

Lifetime social class was classified using the Registrar General's Socioeconomic Classification RGSC.[1] For LBC1936 this was assessed by asking participants what their main qualification was in wave 1 (2004 to 2007). While for the two Twenty-07 cohorts this was assessed by using the highest achieved household social class across all 5 waves of the Twenty-07 study between 1987 and 2008.

Parental social class was classified using RGSC for 1951 (professional, managerial, skilled, semiskilled and unskilled)[2] based on retrospective data. For LBC1936 the measure refers to father's social class at age 11 and was collected at wave 1 (2004/2007). For the Twenty-07 cohorts the measure is for the head of household's social class at age 15 and was measured at wave 1 (1987/1988).

For the Twenty-07 cohorts highest educational qualification (none, standard grades or equivalent, highers or equivalent, HND or equivalent, university degree or equivalent), was collated across all 5 waves of the twenty-07 study from 1987 to 2008. For LBC1936 the highest educational qualification (No qualifications, O-level equivalent, A-level or equivalent, Semi-Professional/Professional qualification, Degree) was assessed at wave 1 (2004 to 2007).

Data on age at first leaving full-time education, recorded in 2007/8 for Twenty-07, and school leaving, recorded in 2004 to 2007 for LBC 1936, were used to calculate minimum school leaving age, which was age 14 for the the1930s cohort, and age 15 for the 1950s cohort and the LBC1936.

Twenty-07 participants were asked what their net income was after tax, but including benefits and pensions, in 2007/8. These scores were then equivalized to account for household composition using McClements scales.[3]

We used participants' datazone of residence to generate SEP measures contemporaneous to Seniors USP in the form of two area deprivation measures, the Scottish Index of Multiple Deprivation 2012 (SIMD)[4] and Carstairs deprivation score.[5] SIMD is a measure of relative deprivation that includes 7 domains including income, employment, education, health, crime and housing, which are used to create an overall deprivation score for each datazone. The Carstairs deprivation score was calculated using the 2011 census and is the sum of four standardised variables the percentage of people living in household with no access to a car or van, percentage of male unemployment, percentage of private households overcrowded, percentage of households with the household reference person in social class IV or V.[6]

Housing tenure (Own or Mortgage versus Renting or Other) was recorded between 2011/2013 for LBC1936 and 2007/8 for 1950s and 1930s cohorts.

In addition, in 2007/8 data for Twenty-07 cohorts, data was collected on car ownership and subjective social status. Car ownership was assessed by asking participants if they or their household owned a car or van. Subjective social status was assessed using a self-anchoring scale in the form of a 10 rung ladder representing society[7] and participants were asked to indicate where they considered themselves to be in relation to others in Britain at the present time.

Appendix table A1: Regression coefficients for the association between SEP measures and sedentary time.

	T07 1950s: Employed		T07 1950s: Retired		T07 1930s: All		LBC1936: All	
	Coef (95% CI)	R^2	Coef (95% CI)	R^2	Coef (95% CI)	R^2	Coef (95% CI)	R^2
**Tenure (Ref = Own/Mortgage)**								
Renting/other	5.42 (-4.76 to 15.59)	0.010	4.52 (0.4 to 8.64)*	0.023	3.04 (-2.16 to 8.24)	0.012	8.61 (2.65 to 14.57)**	0.029
**Car Ownership (Ref = No)**								
Yes	0.26 (-7.51 to 8.03)	0.000	-4.46 (-8.64 to -0.27*)	0.022	-4.57 (-8.68 to -0.47)*	0.041		
**Lifetime Social class (Ref = Professional)**								
II Managerial	1.98 (-3.21 to 7.16)	0.014	1.14 (-2.55 to 4.83)	0.023	6.3 (0.43 to 12.17)*	0.074	2.6 (-0.54 to 5.74)	0.039
III nm Skill non-manual	-1.31 (-8.01 to 5.4)		3.6 (-0.73 to 7.94)		5.76 (-0.51 to 12.03)+		0.39 (-3.34 to 4.12)	
III m Skilled manual	2.7 (-10.93 to 16.33)		5.64 (-1.2 to 12.48)		6.88 (-0.39 to 14.15)+		5.43 (1.37 to 9.49)**	
IV/V Semiskilled / unskilled	0.89 (-21.91 to 23.7)		2.23 (-6.03 to 10.5)		13.46 (4.03 to 22.89)***		6.63 (-0.17 to 13.42)+	
**Highest Qualification (Ref = None)**								
Basic	3.88 (-3.66 to 11.42)	0.012	1.52 (-4.07 to 7.1)	0.034	-5.54 (-10.09 to -0.99)*	0.049	-2.27 (-6.11 to 1.56)	0.008
Professional or Degree	4.4 (-3.56 to 12.37)		-2.42 (-8.04 to 3.2)		-2.69 (-8.36 to 2.98)		-3.02 (-6.98 to 0.94)	
**Minimum School leaving age (Ref = No)**								
Yes	2.59 (-1.82 to 7)	0.013	5.5 (2.54 to 8.46)***	0.064	2.14 (-1.88 to 6.16)	0.010	2.48 (0.01 to 4.96)*	0.014
**Parental social class (Ref = III Skilled)**								
I Professional	1.11 (-8.38 to 10.59)	0.052	-5.11 (-10.41 to 0.18)+	0.098	0.35 (-9.15 to 9.85)	0.039	-0.97 (-5.85 to 3.9)	0.011
II Intermediate Occupations	-4.97 (-10.64 to 0.69)+		-7.43 (-11.39 to -3.48)***		-6.64 (-13.22 to -0.06)*		-0.66 (-3.93 to 2.62)	
IV Partly Skilled manual	-3.86 (-10.29 to 2.57)		2.12 (-1.87 to 6.12)		-0.39 (-6.62 to 5.85)		-2.91 (-7.13 to 1.32)	
V Unskilled	2.72 (-4.31 to 9.76)		-0.17 (-5.46 to 5.12)		-0.74 (-7.75 to 6.27)		-3.14 (-8.68 to 2.4)	
**SIMD**								
Slope Index of Inequality	3.44 (-3.52 to 10.4)	0.009	6.01 (1.56 to 10.46)**	0.035	6.25 (0.35 to 12.15)*	0.036	5.39 (0.5 to 10.27)*	0.017
**Carstairs**								
Slope Index of Inequality	2.39 (-4.98 to 9.76)	0.004	6.35 (1.76 to 10.94)**	0.036	6.51 (0.34 to 12.68)*	0.036	6.84 (1.94 to 11.73)**	0.027
**Gender (Ref= Male)**								
Female	-5.59 (-9.86 to -1.31)*	0.059	-0.85 (-3.83 to 2.13)	0.002	0.5 (-3.49 to 4.5)	0.001	-4.69 (-7.11 to -2.26)***	0.051
**Equivilized Household Income**								
Per £100 increase in Net income	0.15 (-0.57 to 0.86)	0.002	-1.74 (-2.73 to -0.75)***	0.064	-0.51 (-1.52 to 0.5)	0.012		
Per £100 increase income squared			0.08 (0.03 to 0.12)***					
**Subjective social position**								
Each rung on Ladder	1.05 (-0.41 to 2.5)	0.019	-1.46 (-2.3 to -0.63)***	0.058	-1.33 (-2.61 to -0.05)*	0.037		

APPENDIX 2: DOES THE RELATIONSHIP BETWEEN SEDENTARY TIME AND SOCIOECONOMIC POSTION DIFFER BETWEEN WEEKDAYS AND WEEKENDS RESULTS IN THREE OLDER COHORTS

Methods

The analytic sample is the same as that used in the main paper. However, the analytic approach is different. We used a two level multilevel models with days of the week nested within individuals. Daily percentage waking time spent sedentary is the outcome. The SEP measures, type of day (weekday vs weekend) and the interaction between them are the main predictors. Models also adjust for the order of the day in which people participated. i.e. was it the first, second, … or Seventh day of data collection.

All models were estimated using the xtmixed command in Stata 13.1, and the margins post estimation command was used to obtain the results plotted in Figures A1 and A2. The principal question addressed by the analysis is whether there is a different relationship between SEP and sedentary behaviour on weekdays versus weekends and this is indicates by a significant interaction between the SEP measure and the type of day.

Results

The results of the tests for interaction between SEP measures and type of day are shown in appendix table A2. For the 1950s employed group there are significant interaction for six out of the ten SEP measures: SIMD, lifetime social class, subjective Social position, highest qualification and minimum school leaving age. For the retired groups there were between two and four significant interactions, but there was no great consistency in these across the groups.

The results from these models are shown for weekdays in Appendix Figure A1 and for weekends in Appendix Figure A2. For the retired cohorts there is little to suggest there is much in the way of substantive differences between the relationships for weekdays and weekends. For the 1950s employed group, the results are more similar to the retired groups on weekend days than they are during the week. Indeed, for subjective social position and educational qualifications disadvantage may be associated with reduced sedentary behaviour during the week.

Discussion

Our results would suggest that among retired people the association between SEP and sedentary behaviour does not differ between weekends and weekdays. In contrast for employed people different measures of SEP have different associations with sedentary behaviour on weekdays and weekends. The relative small numbers of employed people that we have in the cohort means that these conclusions should be treated with caution.

Appendix Table A2. p values for interaction terms between weekends and SEP measures in the prediction of Sedentary time.

	1950s Employed	1950s Retired	1930s AI	LBC1936	Retired cohorts combined
SIMD	0.020	0.289	0.537	0.035	0.410
Carstairs Deprivation	0.066	0.210	0.947	0.011	0.471
Lifetime Social class RGSC	0.041	0.207	0.021	0.001	0.605
Parental Social Class	0.006	0.063	0.793	0.114	0.850
Income	0.873	0.514	0.756		0.445
Subjective social position	0.000	0.029	0.815		0.047
Highest qualification	0.019	0.487	0.032	0.280	0.514
Minimum School leaving age	0.002	0.957	0.250	0.254	0.835
Tenure	0.322	0.017	0.025	0.971	0.006
Car Ownership	0.384	0.159	0.048		0.019

Caption Appendix Figure A1. Inequalities in weekday sedentary time for three Scottish cohorts.

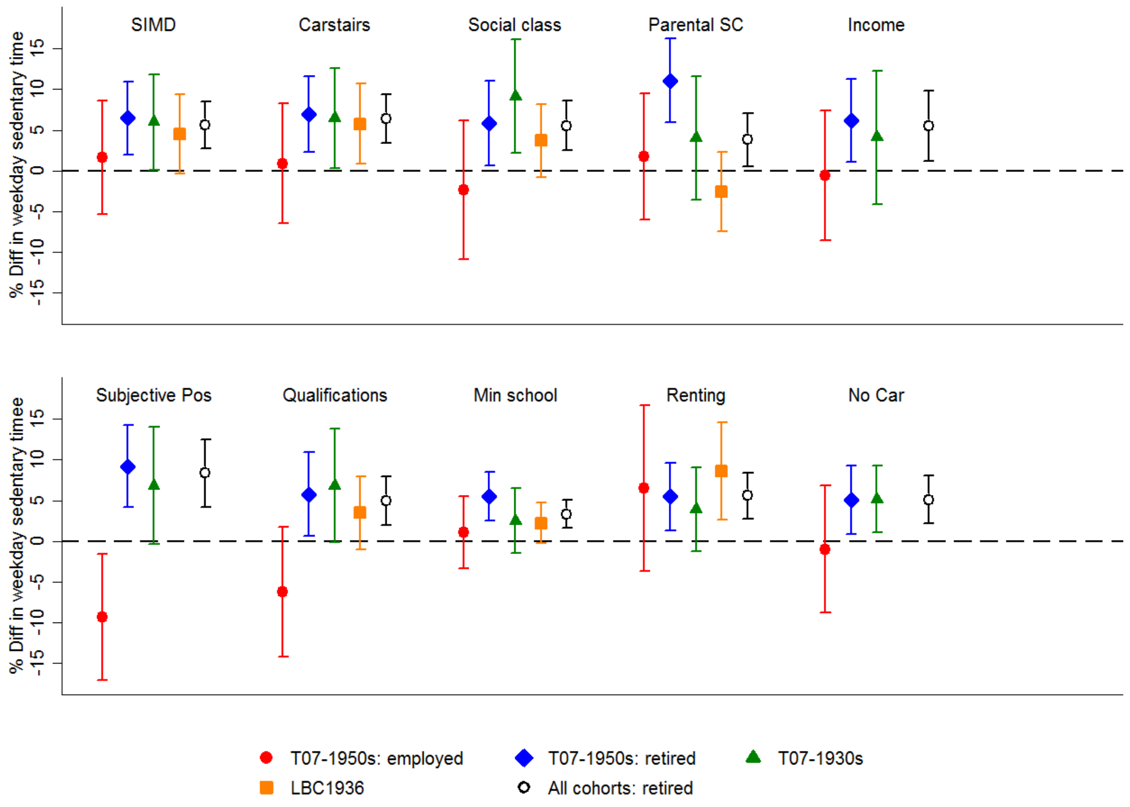

Legend: The inequalities represent SII (with 95% confidence intervals) for each of the following SEP measures (unless otherwise stated in parentheses) the Scottish Index of Multiple Deprivation, Carstairs deprivation score, Lifetime social class, Parental social class, Equivalised household income, Subjective social position, Highest educational qualification, School leaving age (- stayed on beyond minimum reference) Housing tenure (Binary measure –owners reference) and Car ownership (Binary measure – car owners reference). A positive difference (point above the dotted line) indicates that a socioeconomically disadvantaged is more sedentary during the week and a negative difference (point below the line) indicates the reverse.

Caption Appendix Figure A2. Inequalities in weekend sedentary time for three Scottish cohorts.

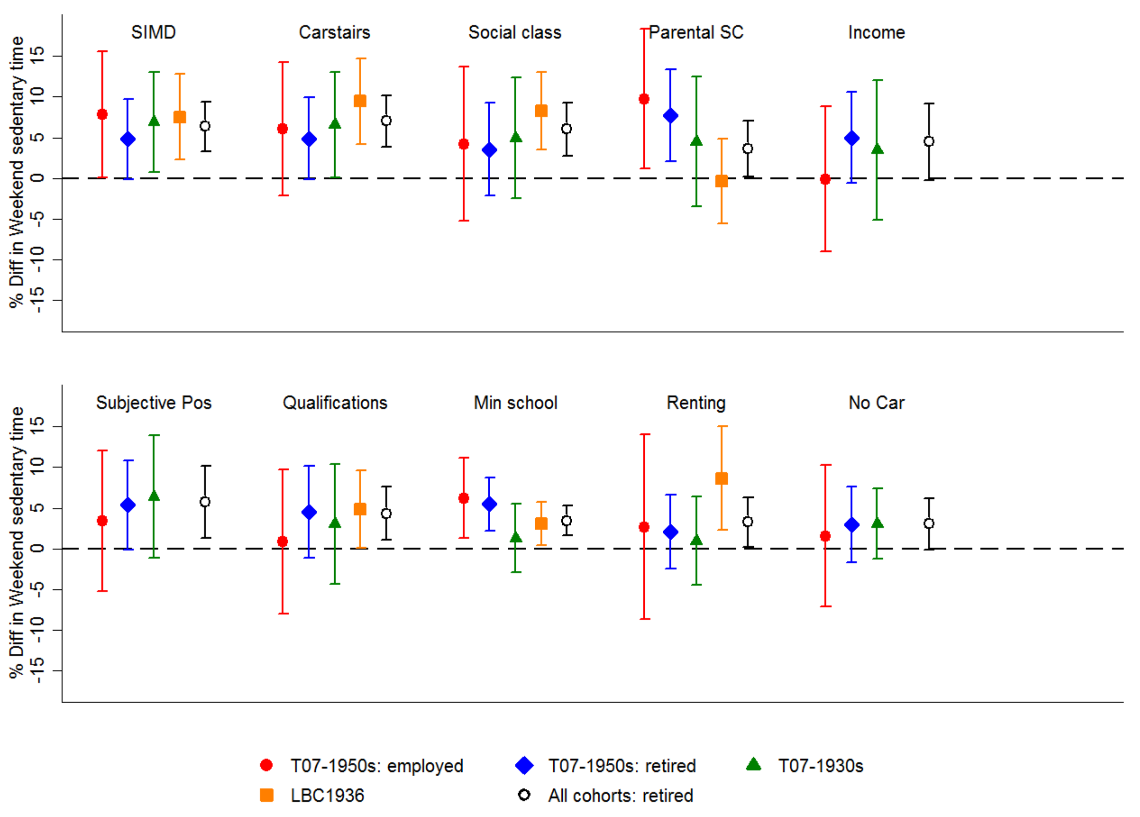

Legend: The inequalities represent SII (with 95% confidence intervals) for each of the following SEP measures (unless otherwise stated in parentheses) the Scottish Index of Multiple Deprivation, Carstairs deprivation score, Lifetime social class, Parental social class, Equivalised household income, Subjective social position, Highest educational qualification, School leaving age (- stayed on beyond minimum reference) Housing tenure (Binary measure –owners reference) and Car ownership (Binary measure – car owners reference). A positive difference (point above the dotted line) indicates that a socioeconomically disadvantaged is more sedentary during the week and a negative difference (point below the line) indicates the reverse.

APPENDIX 3: THE RELATIONSHIP BETWEEN STEP COUNT AND SOCIOECONOMIC POSITION IN OLDER ADULTS IN THREE PROPSECTIVE COHORTS

Methods

The analytical sample and methodology are the same as for the main paper with step count replacing sedentary time as the outcome. Step count was also assessed using the activPAL monitor and was transformed by taking the square root of the data to correct for positive skew. Three people were excluded from the analysis of step count as their step count may have been under recorded due to a shuffling gait.

Results

The mean step counts was as follows for the cohorts 8.9 (SD 3.7) thousand for the Twenty-07 1950s employed cohort, 8.8 (SD 3.9) thousand for the Twenty-07 1950s retired cohort, 5.1 (SD 2.8) thousand for the Twenty-07 1930s cohort and 6.9 (SD 2.8) thousand for LBC1936.

The associations between socioeconomic position and step count are shown in appendix figure 3. The results for step count tend to mirror those for sedentary time but in the opposite direction and with fewer significant relationships. This is not surprising, step count and sedentary time are fairly strongly correlated, the correlations being -0.60, -0.56, -0.51 and -0.48 for the 1950s-employed, 1950s-retired, 1930s cohort and LBC1936 respectively.

Discussion

The results for step count are similar to those for sedentary time. This is not a surprise given the moderately strong correlation. Given that we have used the activPAL data to decompose waking time into time spent sedentary, standing and walking, some correlation between step count and sedentary time is inevitable. Further analysis would benefit from taking a compositional approach modelling the components simultaneously.[8] However, that is beyond the scope of this paper.

Caption Appendix figure 3 Inequalities in Step Count for three Scottish cohorts.

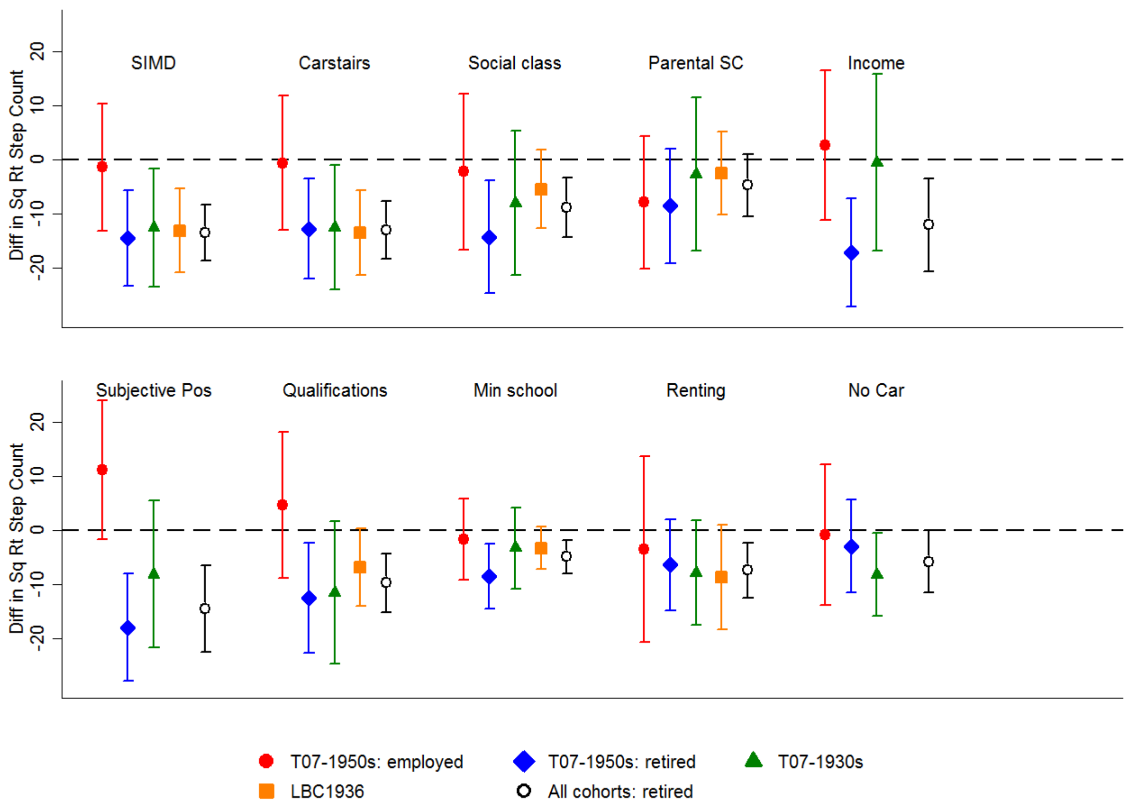

Legend: The inequalities represent SII (with 95% confidence intervals) for each of the following SEP measures (unless otherwise stated in parentheses) the Scottish Index of Multiple Deprivation, Carstairs deprivation score, Lifetime social class, Parental social class, Equivalised household income, Subjective social position, Highest educational qualification, School leaving age (- stayed on beyond minimum reference) Housing tenure (Binary measure –owners reference) and Car ownership (Binary measure – car owners reference). A negative difference (point below the dotted line) indicates that a socioeconomically disadvantaged person makes fewer steps per day a positive difference (point above the line) indicates the reverse.

APPENDIX REFERENCES

1 Office of Population Censuses and Surveys. London: HMSO 1980.

2 Registrar General. The Registrar General's Decennial Supplement, England and Wales 1951, Occupational Mortality Part II, Vol. 1. *Commentary HMSO, London* 1958.

3 McClements LD. Equivalence scales for children. *J Public Econ* 1977;**8**:191-210.

4 Scottish Government. Scottish Index of Multiple Deprivation. 2012.

5 Carstairs V, Morris R. Deprivation: explaining differences in mortality between Scotland and England and Wales. *BMJ* 1989;**299**:886-9.

6 Brown D, Allik M, Dundas R*, et al.* Carstairs Scores for Scottish Postcode Sectors, Datazones and Output Areas from the 2011 Census. MRC/CSO SPHSU, Glasgow. 2014.

7 Singh-Manoux A, Adler NE, Marmot MG. Subjective social status: its determinants and its association with measures of ill-health in the Whitehall II study. *Soc Sci Med* 2003;**56**:1321-33.

8 Chastin SFM, Palarea-Albaladejo J, Dontje ML*, et al.* Combined Effects of Time Spent in Physical Activity, Sedentary Behaviors and Sleep on Obesity and Cardio-Metabolic Health Markers: A Novel Compositional Data Analysis Approach. *PLOS ONE* 2015;**10**:e0139984.
