# Supplementary material for: Relationships between socioeconomic position and objectively measured sedentary behaviour in older adults in three prospective cohorts
Source: BMJ Open. 2017 Jun 15;7(6):e016436. doi: 10.1136/bmjopen-2017-016436 (PMC5541575; doi:10.1136/bmjopen-2017-016436)
Supplement: Author's manuscript [file bmjopen-2017-016436.draft_revisions.pdf]

BMJ Open

Relationships between socioeconomic position and objectively measured sedentary behaviour in older adults in three prospective cohorts

Journal:	*BMJ Open*
Manuscript ID	bmjopen-2017-016436
Article Type:	Research
Date Submitted by the Author:	17-Feb-2017

Complete List of Authors:	Shaw, Richard; University of Glasgow, MRC/CSO Social and Public Health Sciences Unit<br>Čukić, Iva ; University of Edinburgh, Centre for Cognitive Ageing and Cognitive Epidemiology<br>Deary, Ian; University of Edinburgh, Centre for Cognitive Ageing and Cognitive Epidemiology<br>Gale, Catharine; University of Southampton, MRC Lifecourse Epidemiology Unit; University of Edinburgh, Centre for Cognitive Ageing and Cognitive Epidemiology<br>Chastin, Sebastian; Glasgow Caledonian University, School of Health and Life Sciences; Universiteit Gent, Department of Movement and Sports Sciences<br>Dall, Philippa; Glasgow Caledonian University, Institute for applied Health Research, School of Health and Life Sciences<br>Skelton, Dawn; Glasgow Caledonian University, Institute for Applied Health Research, School of Health and Life Sciences<br>Der, Geoff; University of Glasgow, MRC/CSO Social & Public Health Sciences Unit

**Primary Subject Heading**:	Public health
Secondary Subject Heading:	Sociology, Epidemiology
Keywords:	EPIDEMIOLOGY, PUBLIC HEALTH, SOCIAL MEDICINE, SPORTS MEDICINE

SCHOLARONE™ Manuscripts

Relationships between socioeconomic position and objectively measured sedentary behaviour in older adults in three prospective cohorts

Richard J. Shaw[1], Iva Čukić[2], Ian J Deary[2], Catharine R. Gale[2,3], Sebastien F. M. Chastin[4,5], Philippa M. Dall[4], Dawn A. Skelton[4], Geoff Der[1,2] on behalf of the Seniors USP Team

Affiliations

1 MRC/CSO Social and Public Health Sciences Unit, University of Glasgow, Glasgow, United Kingdom.

2 Department of Psychology Centre for Cognitive Ageing & Cognitive Epidemiology, University of Edinburgh, Edinburgh, UK.

3 MRC Lifecourse Epidemiology Unit, University of Southampton, Southampton, UK.

4 Institute for Applied health Research, School of Health and Life Sciences, Glasgow Caledonian University, Glasgow, UK.

5 Department of Movement and Sports Sciences, Faculty of Medicine and Health Science, Ghent University, Ghent, Belgium.

Word count: 2865

Corresponding Author: Richard Shaw
Address: MRC/CSO Social and Public Health Sciences Unit, University of Glasgow,
200 Renfield street, Glasgow, G2 3QB, UK.
Email: richard.shaw@glasgow.ac.uk
Telephone: + 44 (0) 141 353 7565
Fax: + 44 141 337 2389

For peer review only

ABSTRACT

Objectives: Investigate if sedentary behaviour in older adults is associated with a systematic and comprehensive range of socioeconomic position (SEP) measured across the lifecourse. SEP measures included prospective measures of social class, income, educational qualifications and parental social class and contemporaneous measures of area deprivation.

Setting: Glasgow and the surrounding (West of Scotland) combined with Edinburgh and the surrounding area (the Lothians).

Participants: Community dwelling adults aged around 79, 83, and 64 years from, respectively, the Lothian Birth Cohort 1936 (LBC1936) (n=271) and the 1930s (n=119) and 1950s (n=310) cohorts of the West of Scotland Twenty-07 study

Primary outcome measure: Sedentary behaviour was measured objectively using an activPAL activity monitor worn continuously for seven days, and used to calculate percentage of waking time spent sedentary.

Results: Among retired participants, for most cohort and SEP combinations, greater social disadvantage was associated with increased sedentary time. For example, in the Twenty-07 1930s cohort those most deprived on the Carstairs measure spent 6.5% (95% CI 0.3 to 12.7) more of their waking time sedentary than the least deprived. However, for employed people the relationship between SEP and sedentary behaviour was much weaker. For example, the social class difference in percentage waking time sedentary was -0.5% (95% CI -9.0 to 8.0) for employed people in the Twenty-07 1950s cohort as opposed to 5.7% (95% CI 2.6% to 8.7%) for the retired people in all cohorts combined.

Conclusions: Diverse SEP measures were associated with increased sedentary behaviour among retired people. There was little evidence for a relationship among employed older adults.

For peer review only - http://bmjopen.bmj.com/site/about/guidelines.xhtml

Strengths and limitations of this study

- Sedentary behaviour is objectively measured using an activPAL monitor that correctly identifies posture.
- Data is available for an entire seven day period thus minimising any systematic variation over the course of the week.
- Our study is the first that we are aware of that uses a comprehensive and systematic range of SEP measures across the lifecourse.
- Due to attrition the sample is more socioeconomically advantaged than the original populations from which it was drawn.

For peer review only

For peer review only - http://bmjopen.bmj.com/site/about/guidelines.xhtml

Sedentary behaviour, defined as energy expenditure ≤ 1.5 metabolic equivalents (METs) while awake and in a sitting or reclining posture,[1] is emerging as a modifiable risk factor for poor health independent of physical activity.[2, 3] There is evidence for associations with: mortality,[3-5] cancer incidence,[3] diabetes,[6] bone density[7] and falls.[8] Sedentary behaviour increases with age.[9] On average, sedentary time represents 65–80 % of an older adult's waking day[10] and 67 % of older adults spend in excess of 8.5 h per day sitting.[11] Reducing sedentary behaviour may, therefore, be an important health message for older adults.

Socio economic position (SEP) has been described as a fundamental cause of poor health and health inequalities.[12] SEP represents flexible resources that shape people's opportunities and provide support for their efforts to engage health enhancing behaviours.[12] As such, it is a multi-dimensional concept[13] with different aspects of socio-economic position being salient for different health outcomes and the importance of those aspects varying across the life course.[14]

The current literature on the socioeconomic determinants of sedentary behaviour in older adults is very limited.[9, 15] Of the few studies that exist most rely on self-reported measures of TV viewing and screen time.[16-22] However, these represent only two of the circumstances in which people might be sedentary. Moreover, self-reported measures of sedentary behaviour have only low to moderate validity[23] and, therefore, attenuate relationships. They are also subject to recall and social desirability bias.

To our knowledge, only four studies have examined the association of SEP with objectively measured sedentary behaviour in older people and these have all used ActiGraph accelerometers worn on the waist,[24-27] which do not accurately record posture.[28] Moreover, these studies did not explicitly focus on SEP as a potential determinant of sedentary behaviour; SEP was simply treated as a confounder and the SEP measures used were relatively crude. In short, while results from younger adults suggest SEP could be an important predictor of sedentary behaviour,[29] the situation for older adults remains unclear.

In this study we investigate whether SEP is an important determinant of sedentary behaviour among older people. We use data from three Scottish cohorts aged in their sixties, seventies and eighties. Sedentary behaviour is measured using the activPAL monitor worn continuously for seven days. As the participants are drawn from existing, longstanding cohorts, our study includes a diverse range of prospective indicators that capture many aspects of socio-economic position including parental social class, education, household social class, neighbourhood deprivation, housing tenure, income and subjective social status.

METHODS

Participants

This study, Seniors USP (Understanding Sedentary Patterns), comprises subsamples of the Lothian Birth Cohort 1936 (LBC1936) and the West of Scotland Twenty-07 study (Twenty-07). Full details for these studies are available elsewhere.[30, 31] The Twenty-07 study itself comprises three age cohorts although only the two oldest are included here (hereafter, referred to as the 1930s and 1950s cohorts according to their decade of birth). Data for the main Twenty-07 study were collected

For peer review only - http://bmjopen.bmj.com/site/about/guidelines.xhtml

in five waves of interviews between 1987 and 2008. LBC1936 is an on-going cohort study of older people that began in 2004 as a follow up to the Scottish Mental Survey 1947.

Data specific to this study, including objective sedentary behaviour, were collected between November 2014 and April 2016 when the mean ages of the cohorts were around 64, 79 and 83 years.

To be eligible, participants had to live in the community and needed sufficient cognitive ability to be able to provide informed consent and also a sufficiently good memory to complete sleep diaries. Beyond these minimum requirements people were not excluded due to physical or mental impairments. Twenty-07 participants were eligible if they lived within the greater Glasgow area. All eligible people in the 1930s cohort were approached and a random sample of eligible people in the 1950s cohort was selected. Consecutive recruits to wave 4 of LBC1936 were invited to join Seniors USP until the target sample size of 300 was achieved.

For peer review only

Sedentary behaviour

Sedentary behaviour was measured using the activPAL monitor (activPAL3c, PAL Technologies Ltd, Glasgow, UK) which provides accurate and reliable measurement of sedentary behaviour.[32, 33] The device is small and light (53x35x7mm; 15g) and was worn attached to the anterior thigh of the dominant leg with a waterproof dressing. Participants were initially interviewed for basic socio-demographic and health information and were then asked to wear the activPAL continuously for seven days, including overnight and during bathing/swimming, while going about their usual daily activities. Participants also kept a diary reporting the time they fell asleep the previous night and the time they woke up for each day of monitoring.

The outcome measure is the percentage of waking time spent sedentary, averaged over the seven days (hereafter, sedentary time).

Socio-Economic Position

We include prospective measures of SEP based on the three major axes of social stratification: education, occupation and income. In addition there are two measures of area deprivation, and one measure each of tenure, car ownership and subjective social position. Full details of these measures are given in the appendix. A brief description follows.

Occupation based measures are parental social class (Professional/intermediate/skilled/semi-skilled/unskilled) and lifetime social class (Professional/managerial/skilled non-manual/skilled manual/semi-skilled or unskilled).

Education measures are: highest educational qualification (none/basic/degree or professional) and whether or not left school at minimum leaving age.

The income measure is net household income equivalised to adjust for household composition using the McClements scales.[34]

For peer review only - http://bmjopen.bmj.com/site/about/guidelines.xhtml

Area deprivation measures include the Scottish Index of Multiple Deprivation 2012 (SIMD)[35] and Carstairs deprivation score.[36] Both measures are based on the datazone of the participant's residence. The SIMD comprises seven domains: income, employment, education, health, crime, housing and access to facilities which are combined to create an overall deprivation score. The Carstairs deprivation score is based on four measures from the 2011 census: car ownership, male unemployment, overcrowding and low social class.[37]

Our measure of Housing tenure contrasts home owners with others and likewise our measure of car ownership.

Subjective social status was assessed using a self-anchoring scale in the form of a 10 rung ladder representing society[38] and participants were asked to indicate where they considered themselves to be in relation to others in Britain.

Statistical Methods

Differences between the cohorts were identified using Chi-square tests for the ordinal and binary measures and Anova for the continuous measures. Associations between SEP and outcome measures were estimated using linear regression.

In order to facilitate comparisons between SEP measures made on different scales of measurement we used the slope index of inequality (SII).[39] This involves rescaling the SEP measures to fractional ranks, that is, ranking them and dividing by the sample size. Where there are ties in the data the mid-rank is assigned. For the area deprivation measures, ranks are available for the whole of Scotland. For other measures, ranking is cohort specific. For highest educational qualifications, all 5 ordinal categories (see appendix) that were available for each cohort were used to derive the SII, while three categories were used for presentation in tables. For all measures, higher ranks are assigned to greater disadvantage and the SII can therefore be interpreted as the difference in outcome between the hypothetically most and least disadvantaged.

Additional analyses based on the original scores are presented in the appendix.

All analyses were conducted using Stata version 13.1.

RESULTS

Seven hundred and sixty two participants took part: 340, 129 and 304 each from the 1950s cohort, 1930s, and LBC1936, respectively. Of these, 700 (91%) provided seven full days of activPAL and sleep diary data.

Previous research[40] has suggested that the social patterning of physical activity differs before and after retirement and preliminary analysis of sedentary behaviour in the 1950s cohort showed a similar pattern. Consequently we have divided this cohort into those still employed, including the semi-retired, vs those no longer employed. We refer to the latter as 'retired' even though not all would consider themselves formally retired.

Table 1 shows the breakdown of the sample by SEP and demographic measures. There were significant cohort differences in all the SEP measures (p<.01), except parental social class, reflecting

For peer review only - http://bmjopen.bmj.com/site/about/guidelines.xhtml

Table 1: Descriptive statistics by cohort and employment status during Seniors USP

	Twenty-071950s Employed n=110		Twenty-07 1950s Retired n=200		T07 1930s All n=119		LBC 1936 All n=271	
	n	%	n	%	n	%	n	%
**Tenure**								
Own or Mortgage	104	94.6	171	85.5	95	79.8	259	95.6
Renting or Other	5	4.6	28	14.0	21	17.7	12	4.4
Missing	1	0.9	1	0.5	3	2.5	0	0.0
**Car Ownership**								
No	9	8.2	27	13.5	42	35.3		
Yes	100	90.9	172	86.0	74	62.2		
Missing	1	0.9	1	0.5	3	2.5		
**Lifetime Social class RGSC**								
I Professional	27	24.6	45	22.5	18	15.1	69	25.5
II Managerial	60	54.6	94	47.0	47	39.5	101	37.3
III Skilled non manual	19	17.3	43	21.5	31	26.1	50	18.5
III Skilled manual	3	2.7	11	5.5	16	13.5	38	14.0
IV/V Semi-skilled un skilled	1	0.9	7	3.5	7	5.5	10	3.7
Missing	0	0.0	0	0.0	0	0.0	3	1.1
**Highest qualification**								
None	10	9.1	15	7.5	34	28.6	36	13.3
Basic	65	59.1	96	48.0	61	51.3	133	49.1
Degree or Professional qualification	35	31.8	89	44.5	24	20.2	102	37.6
**Minimum School leaving age**								
Stayed past min age	69	62.7	131	65.5	61	51.3	144	53.1
Left minimum or earlier	40	36.4	68	34.0	55	46.2	127	46.9
Missing	1	0.9	1	0.5	3	2.5	0	0
**Parental Social Class**								
I Professional	6	5.5	16	8.0	6	5.0	20	7.4
II Intermediate	21	19.1	33	16.5	14	11.8	54	19.9
III skilled (manual & non-manual)	54	49.1	98	49.0	61	51.3	136	50.2
IV Partly Skilled	15	13.6	32	16.0	16	13.5	28	10.3
V Un skilled	12	10.9	16	8.0	12	10.1	15	5.5
Missing	2	1.8	5	2.5	10	8.4	18	6.6
**Gender**								
Male	70	63.6	75	37.5	54	45.4	140	51.7
Female	40	36.4	125	62.5	65	54.6	131	48.3
**SIMD**								
Mean	0.37		0.42		0.45		0.22	
SD	0.30		0.32		0.33		0.25	
**Carstairs**								
Mean	0.39		0.44		0.47		0.3	
SD	0.29		0.31		0.32		0.25	
**Equivalized Household net Income /£100**								
Mean	5.78		6.22		4.01			
SD	3.09		3.92		2.39			
Missing	11		15		22			
**Subjective social potion**								
Mean	6.52		6.14		6.1			
SD	1.47		1.70		1.62			
Missing	2		3		7			
**Age**								
Mean	64.4		64.7		83.4		79.0	
SD	0.88		0.89		0.62		0.44	

For peer review only - http://bmjopen.bmj.com/site/about/guidelines.xhtml

differences between the areas from which they are drawn and secular changes in the occupational structure. For all SEP measures the 1930s cohort are the most disadvantaged.

Within the 1950s cohort there were few differences in SEP measures between the employed and retired. The only significant differences were for gender (0.001), only 36.4% of 1950s employed were female compared to 62.5% of the retired, and housing tenure for which 14.0% of the retired were renting compared to only 4.6% of the employed.

Table 2 shows the mean and standard deviation of sedentary time by cohort. As might be expected given their ages, the 1930s cohort are the most sedentary, on average spending 68.2% of the day sedentary. Among the 1950s cohort the retired have similar levels to LBC1936 (62.2% and 62.5%, respectively) whereas the employed have the lowest level (58.3%). Despite LBC1936 being closer in age to the 1930s cohort, their sedentary time is more like the 1950s retired. As the 1930s cohort is the most deprived and LBC1936 the least, it may be that the overall cohort differences reflect the differences in SEP as well as age differences.

Throughout the table there are numerous examples of SEP differences in sedentary time although the small numbers in some categories, especially the extremes of some social class measures, need to be borne in mind.

A comparison of the analysed sample with the remainder of those invited showed a number of differences (data not shown). For LBC1936 there were no significant differences in lifetime and parental social class, gender or self-rated health, but the analysed sample were more advantaged with respect to tenure, educational qualifications and minimum school leaving age. For the 1950s cohort the analysed sample were more advantaged with respect to all the prospective SEP measures and self-rated health but there was no difference in gender. For the 1930s cohort the analysed sample had better self-rated health and older school leaving age, but there were no significant differences for the other SEP measures.

Analysis of Sedentary time

Figure 1 presents the results of linear regression analyses regressing sedentary time on the SEP measures. Separate results are shown for: the LBC1936 cohort; the 1930s cohort; the employed and retired subgroups of the 1950s cohort; and the 1950s retired group combined with the two older cohorts. In each case, regression coefficients and their 95% confidence intervals are shown. For the binary SEP measures the effect is simply the difference in sedentary time between the two groups. For the other SEP measures the effect is the SII and is interpretable as the difference in sedentary time between the most and least deprived.

The overall pattern is one of more disadvantaged SEP being associated with greater sedentary time. The exceptions are the employed group and parental social class where there are no clear or consistent patterns. In terms of statistical significance, when the retired people in the 1950s cohort was combined with the two older cohorts all SEP measures were significantly and positively related to sedentary time, the largest SIIs being found for subjective social position (7.6% CI 3.5 to 11.7) and Carstairs deprivation (6.6 % CI 3.6 to 9.5). When these cohorts were investigated separately, relationships between sedentary time and SEP were found for most cohort and SEP combinations. In some cases, particularly for the 1930s cohort, the confidence intervals crossed zero although effects were in the same direction and of similar magnitude.

For peer review only - http://bmjopen.bmj.com/site/about/guidelines.xhtml

Table 2: Mean and standard deviation for and percent waking time spent sedentary

	Twenty07 1950s Employed		Twenty07 1950s Retired		Twenty07 1930s All		LBC1936 All	
	Mean	SD	Mean	SD	Mean	SD	Mean	SD
**All members**								
Overall	58.3	11.2	62.2	10.3	68.2	10.9	62.5	10.4
**Tenure**								
Own or Mortgage	58.1	11.4	61.5	10.1	67.8	10.8	62.1	10.2
Renting or Other	63.5	6.6	66.1	10.9	70.8	11.3	70.7	11.9
**Car Ownership**								
No	58.1	11.5	66.0	9.7	71.2	10.7		
Yes	58.4	11.2	61.6	10.3	66.7	10.8		
**Lifetime social class**								
I Professional	57.4	13.3	60.5	9.7	62.5	11.2	60.3	10.1
II Managerial	59.5	10.2	61.6	10.4	68.8	11.5	62.9	10.0
III Skilled non manual	56.1	11.2	64.1	9.7	68.3	9.4	60.7	10.5
III Skilled manual	60.1	14.7	66.2	12.6	69.4	10.0	65.7	10.8
IV/V Semi-skilled un skilled	58.3	-	66.7	13.0	76.0	10.7	66.9	8.9
**Highest achieved qualification**								
None	54.7	13.9	62.6	11.4	71.6	10.0	64.8	11.7
Basic	58.5	11.0	64.1	9.9	66.1	11.4	62.5	10.2
Degree/Prof qualification	59.1	10.8	60.1	10.4	68.9	10.2	61.7	10.1
**Minimum School leaving age**								
Stayed pass min age	57.4	11.4	60.3	9.8	67.3	10.2	61.3	10.1
Left minimum or earlier	60.0	10.8	65.8	10.5	69.4	11.6	63.8	10.5
**Parental Social Class**								
I Professional	60.7	11.8	58.4	9.9	69.7	7.7	66.5	7.2
II Intermediate	54.6	13.1	56.1	9.8	62.7	12.3	62.2	9.4
III skilled (manual & non-manual)	59.6	10.1	63.5	10.2	69.4	5.0	62.5	9.6
IV Partly Skilled	55.7	11.5	65.6	10.0	69.0	14.1	63.2	10.4
V Un skilled	62.3	11.0	63.3	8.8	68.6	15.8	60.3	11.8
**Gender**								
Male	60.4	10.9	62.7	9.1	68.0	12.0	64.8	9.8
Female	54.8	10.8	61.9	11.0	68.5	10.0	60.1	10.5
**SIMD Overall**								
<50 Percentile	57.4	11.4	60.4	9.6	67.0	10.6	61.9	10.2
> 50 Percentile	59.4	10.9	63.9	10.7	69.4	11.2	63.1	10.5
**Carstairs Score**								
< 50 Percentile	57.7	11.8	60.4	9.5	66.8	11.1	60.9	9.6
> 50 Percentile	59.1	10.4	63.8	10.8	69.6	11.6	64.2	10.9
**Income**								
> 50 Percentile	57.6	11.5	60.7	10.0	67.2	11.8		
< 50 Percentile	59.0	10.6	64.0	10.6	71.1	10.4		
**Subjective social position**								
> 50 Percentile	59.5	11.0	60.2	9.3	66.4	10.6		
< 50 Percentile	57.2	11.5	64.5	11.0	69.3	11.4		

For peer review only - http://bmjopen.bmj.com/site/about/guidelines.xhtml

In contrast to the retired cohorts, none of the SEP measures were significantly associated with sedentary time for the employed in the 1950s cohort. Furthermore half of the coefficients were below zero suggesting that there was no evidence of a relationship between sedentary time and SEP measures in general.

Additional analyses are presented in the appendix. Table A1 includes the regression coefficients for the SEP measures in their original form as shown in Tables 1 and 2. Broadly these are consistent with the results produce by the SII; however, far fewer measures are significant, partly reflecting the reduced statistical power of categorical variables. It should also be noted that there was a non-linear relationship between income and sedentary time among the retired subgroup of the 1950s cohort. This indicates that sedentary time falls with increases in net equivalized household incomes up to about £1,000 a month beyond which further income makes little difference.

DISCUSSION

Among older, retired adults we find consistent evidence that socially disadvantaged people with respect to nearly all SEP measures are more sedentary than their advantaged counterparts. In contrast there was little evidence of a relationship between SEP and sedentary time for those still employed.

We identified only four previous studies that measured sedentary behaviour objectively in older adults and included measures of SEP.[24-27] All of these used ActiGraph accelerometers worn on the hip which are unable to consistently distinguish sitting from standing.[28] None of the studies focused specifically on SEP as a determinant of SB. Van Holle et al. included measures of education, occupation and income but did not examine their association with sedentary behaviour.[25] The other three studies present a mixed picture. Of the two that included a measure of education, higher educational attainment was significantly associated with lower sedentary time in one[27] whilst the more educated were more sedentary in the other,[29] although the significance of the latter was not clear. Two studies included occupation or social class[24, 27] but there was no significant association with sedentary behaviour in either. One study included income which was not significantly associated with sedentary behaviour.[26]

Aside from the different measurement of sedentary behaviour there are possible explanations for these differences. In the study by Dunlop et al.[26] 47% of the NHANES sample were aged 60-69 so a large proportion would still be employed and, if the pattern observed here is true of the USA, this might obscure the relationship amongst the retired. In the study by van der Berg et al.[27] the occupational classification was relatively crude with little discrimination amongst the women, 69% of whom were homemakers. Sartini et al.[24] used a dichotomised social class as a covariate in mutually adjusted models containing a large number of variables several of which might plausibly lie on the causal pathway.

Investigations of the influence of neighbourhood SEP among older adults is limited to studies of self-reported sedentary behaviour in which neighbourhoods have been dichotomised into low and high income.[17, 21] We are not aware of any studies investigating the influence of housing tenure, car ownership or subjective social position on sedentary time.

For peer review only - http://bmjopen.bmj.com/site/about/guidelines.xhtml

The most important result of our study is that social inequalities in sedentary behaviour are greater in retired people than among the employed. Studies of working age adults such as Van Dyck et al (2010),[29] suggest that white collar workers may in fact be more sedentary. Other research has shown that on retirement manual workers lose activity gained from work which is not compensated for by increasing leisure time activity.[40]

We find relationships even with measures set relatively early in life such as school leaving age, suggesting that the pathways between SEP and sedentary behaviour may be established early in life.

Strengths and limitations

Our study has the most comprehensive range of SEP measures in any investigation of sedentary behaviour that we are aware of. The measures are made at the individual, household and area level and pertain to different stages throughout the lifecourse, some quite distal to the outcomes. The area level measures are obtained by geocoding the participant's residential address and so are objective. Even those that were self-reported were ascertained prior to, and independently of, the measurement of sedentary behaviour. Finding consistent results across such a wide range of measures suggests that our results are unlikely to be due to chance.

We used the activPAL3 monitor which provides an objective measure of sedentary behaviour that correctly identifies posture. The activPAL is also worn continuously whereas hip worn monitors are typically removed at night, and when showering or bathing, which introduces additional sources of error.

The data cover an entire seven day period thus minimising any systematic variation over the course of the week. A full week of data was available for a very high proportion of the participants.

We used the slope index of inequality to facilitate comparison of SEP indicators made on different scales.

Sampling from existing cohorts also has its drawbacks. As longitudinal studies progress they increasingly suffer from attrition and survival bias. Furthermore, the sample included here is generally more advantaged than the cohorts from which they were drawn. However, it is unclear whether this will have biased the relationships estimated here.

Another weakness of our approach is the reliance on self-reports of sleep and waking times. Efforts to accurately identify sleep time from accelerometry data might prove fruitful for future research.

CONCLUSIONS

In conclusion, sedentary behaviour appears to be socially patterned among older people after retirement but not before. The results here reinforce the message that retirement is a key transition and an opportunity for interventions to improve health and lessen health inequalities. Policies to address health inequalities in later life should provide opportunities and support for older people to develop habits and leisure time activities that replace sedentary behaviour.

For peer review only - http://bmjopen.bmj.com/site/about/guidelines.xhtml

For peer review only

ACKNOWLEDGEMENTS

The named authors present the study on behalf of the Seniors USP Team, which comprises: Dawn A Skelton (PI), Sebastien Chastin, Simon Cox, Elaine Coulter, Iva Čukić, Philippa Dall, Ian Deary, Geoff Der, Manon Dontje, Claire Fitzsimons, Catherine Gale, Jason Gill, Malcolm Granat, Cindy Gray, Carolyn Greig, Elaine Hindle, Karen Laird, Gillian Mead, Nanette Mutrie, Victoria Palmer, Ratko Radakovic, Naveed Sattar, Richard Shaw, John Starr, Sally Stewart, Sally Wyke. The Seniors USP (understanding sedentary patterns) project is funded by the UK Medical Research Council (MRC) as part of the Lifelong Health and Wellbeing Initiative (LLHW) [MR/K025023/1].

The Lothian Birth Cohort 1936 (LBC1936) thank the cohort members, investigators, research associates and team members. We also thank the radiographers at the Brain Research Imaging Centre, and the research nurses and Genetics Core staff at the Wellcome Trust Clinical Research Facility. LBC1936 data collection are supported by the Disconnected Mind project (funded by Age UK and MRC [Mr/M01311/1 and G1001245/96077]) and undertaken within the University of Edinburgh Centre for Cognitive Ageing and Cognitive Epidemiology (funded by the BBSRC and MRC as part of the LLHW [MR/K026992/1]).

The West of Scotland Twenty-07 Study was funded by the MRC and the data were originally collected by the MRC Social and Public Health Sciences Unit (MC_A540_53462). We thank all of the cohort participants, and the survey staff and research nurses who carried it out. The data are employed here with the permission of the Twenty-07 Steering Committee.

ETHICS

Ethics approval for the Twenty-07 West of Scotland study participants was gained for each wave from the NHS and / or Glasgow University Ethics Committees. Ethical approval for LBC1936 was obtained from the Multi-Centre Research Ethics Committee and from Lothian Research Ethics Committee. All participants gave written, informed consent.

DATA SHARING AGREEMENT

Data collected as part of the Seniors USP study is embargoed until October 2018. Thereafter data sharing will be governed by the agreements already in place for the Twenty-07 and LBC1936 studies.

The West of Scotland Twenty-07 study is managed by the MRC/CSO Social and Public Health Sciences Unit, University of Glasgow. Further information about how to access the data can be found at http://2007study.sphsu.mrc.ac.uk/

Data are available upon request from the Lothian Birth Cohort 1936 Study. To request the data readers should contact the principal investigator, Ian Deary, who can be contacted at i.deary@ed.ac.uk.

For peer review only

AUTHOR CONTRIBUTIONS

SFMC, PMD, IJD, GD, CRG and DAS contributed to the design of the study. GD conceived original idea for the paper and provided statistical support. RJS conducted the analyses and wrote the first draft of the manuscript. IC assisted with preparation of LBC1936 data. IJD is director of the LBC1936 study. CRG provided statistical advice. IC, IJD, CRG drafted LBC1936 methodology. PMD and SFMC contributed to acquisition, analysis and processing of activPAL data. DAS was the principle investigator for Seniors USP and provided gerontological advice. All authors have read and commented on the manuscript and approved the final version.

COMPETING INTERESTS

ID is supported by Age UK. PD has received grant funding from PAL technologies outside the submitted work.

For peer review only

For peer review only - http://bmjopen.bmj.com/site/about/guidelines.xhtml

REFERENCES

1 Sedentary Behaviour Research Network. Letter to the Editor: Standardized use of the terms "sedentary" and "sedentary behaviours". *Appl Physiol Nutr Metab* 2012;**37**:540-2.

2 Owen N, Sugiyama T, Eakin EE*, et al.* Adults' Sedentary Behavior: Determinants and Interventions. *Am J Prev Med* 2011;**41**:189-96.

3 Biswas A, Oh PI, Faulkner GE*, et al.* Sedentary time and its association with risk for disease incidence, mortality, and hospitalization in adults: A systematic review and meta-analysis. *Ann Intern Med* 2015;**162**:123-32.

4 Katzmarzyk PT, Church TS, Craig CL*, et al.* Sitting time and mortality from all causes, cardiovascular disease, and cancer. *Med Sci Sports Exerc* 2009;**41**:998-1005.

5 Hagger-Johnson G, Gow AJ, Burley V*, et al.* Sitting Time, Fidgeting, and All-Cause Mortality in the UK Women's Cohort Study. *Am J Prev Med* 2016;**50**:154-60.

6 Wilmot EG, Edwardson CL, Achana FA*, et al.* Sedentary time in adults and the association with diabetes, cardiovascular disease and death: systematic review and meta-analysis. *Diabetologia* 2012;**55**:2895-905.

7 Chastin SFM, Mandrichenko O, Helbostadt JL*, et al.* Associations between objectively-measured sedentary behaviour and physical activity with bone mineral density in adults and older adults, the NHANES study. *Bone* 2014;**64**:254-62.

8 Jefferis BJ, Merom D, Sartini C*, et al.* Physical Activity and Falls in Older Men: The Critical Role of Mobility Limitations. *Med Sci Sports Exerc* 2015;**47**:2119-28.

9 Chastin S, Buck C, Freiberger E*, et al.* Systematic literature review of determinants of sedentary behaviour in older adults: a DEDIPAC study. *Int J Behav Nutr Phys Act* 2015;**12**:127.

10 Harvey JA, Chastin SFM, Skelton DA. How Sedentary Are Older People? A Systematic Review of the Amount of Sedentary Behavior. *J Aging Phys Act* 2015;**23**:471-87.

11 Harvey J, Chastin S, Skelton D. Prevalence of Sedentary Behavior in Older Adults: A Systematic Review. *Int J Environ Res Public Health* 2013;**10**:6645-61.

12 Phelan JC, Link BG, Tehranifar P. Social Conditions as Fundamental Causes of Health Inequalities: Theory, Evidence, and Policy Implications. *J Health Soc Behav* 2010;**51**:S28-S40.

13 Krieger N, Williams DR, Moss NE. Measuring Social Class in US Public Health Research: Concepts, Methodologies, and Guidelines. *Annu Rev Public Health* 1997;**18**:341-78.

14 Galobardes B, Shaw M, Lawlor DA*, et al.* Indicators of socioeconomic position (part 1). *J Epidemiol Community Health* 2006;**60**:7-12.

15 Brug J, Chinapaw M. Determinants of engaging in sedentary behavior across the lifespan; lessons learned from two systematic reviews conducted within DEDIPAC. *Int J Behav Nutr Phys Act* 2015;**12**:134.

16 Van Cauwenberg J, De Donder L, Clarys P*, et al.* Relationships of individual, social, and physical environmental factors with older adults' television viewing time. *J Aging Phys Act* 2014;**22**:508-17.

17 Ding D, Sugiyama T, Winkler E*, et al.* Correlates of Change in Adults' Television Viewing Time: A Four-Year Follow-up Study. *Med Sci Sports Exerc* 2012;**44**:1287-92.

18 Smith L, Gardner B, Hamer M. Childhood correlates of adult TV viewing time: a 32-year follow-up of the 1970 British Cohort Study. *J Epidemiol Community Health* 2014.

19 Gardner B, Iliffe S, Fox K*, et al.* Sociodemographic, behavioural and health factors associated with changes in older adults' TV viewing over 2years. *Int J Behav Nutr Phys Act* 2014;**11**:102.

20 Busschaert C, Scherrens A-L, De Bourdeaudhuij I*, et al.* Socio-Ecological Variables Associated with Context-Specific Sitting Time in Belgian Older Adults: A One-Year Follow-Up Study. *PLOS ONE* 2016;**11**:e0167881.

21 Sugiyama T, Salmon J, Dunstan DW*, et al.* Neighborhood Walkability and TV Viewing Time Among Australian Adults. *Am J Prev Med* 2007;**33**:444-9.

For peer review only - http://bmjopen.bmj.com/site/about/guidelines.xhtml

22 Clark BK, Sugiyama T, Healy GN, *et al.* Socio-demographic correlates of prolonged television viewing time in Australian men and women: the AusDiab study. *J Phys Act Health* 2010;**7**:595-601.
23 Celis-Morales CA, Perez-Bravo F, Ibañez L, *et al.* Objective vs. Self-Reported Physical Activity and Sedentary Time: Effects of Measurement Method on Relationships with Risk Biomarkers. *PLOS ONE* 2012;**7**:e36345.
24 Sartini C, Wannamethee S, Iliffe S, *et al.* Diurnal patterns of objectively measured physical activity and sedentary behaviour in older men. *BMC Public Health* 2015;**15**:609.
25 Van Holle V, Van Cauwenberg J, De Bourdeaudhuij I, *et al.* Interactions between Neighborhood Social Environment and Walkability to Explain Belgian Older Adults' Physical Activity and Sedentary Time. *Int J Environ Res Public Health* 2016;**13**:569.
26 Dunlop DD, Song J, Arntson EK, *et al.* Sedentary time in US older adults associated with disability in activities of daily living independent of physical activity. *J Phys Act Health* 2015;**12**:93.
27 van der Berg JD, Bosma H, Caserotti P, *et al.* Midlife determinants associated with sedentary behavior in old age. *Med Sci Sports Exerc* 2014;**46**:1359-65.
28 Crouter SE, Clowers KG, Bassett DR. A novel method for using accelerometer data to predict energy expenditure. *J Appl Physiol* 2006;**100**:1324-31.
29 Van Dyck D, Cardon G, Deforche B, *et al.* Neighborhood Walkability and Sedentary Time in Belgian Adults. *Am J Prev Med* 2010;**39**:25-32.
30 Deary IJ, Gow AJ, Pattie A, *et al.* Cohort Profile: The Lothian Birth Cohorts of 1921 and 1936. *Int J Epidemiol* 2012;**41**:1576-84.
31 Benzeval M, Der G, Ellaway A, *et al.* Cohort Profile: West of Scotland Twenty-07 Study: Health in the Community. *Int J Epidemiol* 2009;**38**:1215-23.
32 Kozey-Keadle S, Libertine A, Lyden K, *et al.* Validation of wearable monitors for assessing sedentary behavior. *Med Sci Sports Exerc* 2011;**43**:1561-7.
33 Sellers C, Dall P, Grant M, *et al.* Validity and reliability of the activPAL3 for measuring posture and stepping in adults and young people. *Gait Posture* 2016;**43**:42-7.
34 McClements LD. Equivalence scales for children. *J Public Econ* 1977;**8**:191-210.
35 Scottish Government. Scottish Index of Multiple Deprivation. 2012.
36 Carstairs V, Morris R. *Deprivation: explaining differences in mortality between Scotland and England and Wales* 1989.
37 Brown D, Allik M, Dundas R, *et al.* Carstairs Scores for Scottish Postcode Sectors, Datazones and Output Areas from the 2011 Census. 2014.
38 Singh-Manoux A, Adler NE, Marmot MG. Subjective social status: its determinants and its association with measures of ill-health in the Whitehall II study. *Soc Sci Med* 2003;**56**:1321-33.
39 Wagstaff A, Paci P, van Doorslaer E. On the measurement of inequalities in health. *Soc Sci Med* 1991;**33**:545-57.
40 Berger U, Der G, Mutrie N, *et al.* The impact of retirement on physical activity. *Ageing Soc* 2005;**25**:181-95.

For peer review only

For peer review only - http://bmjopen.bmj.com/site/about/guidelines.xhtml

Caption Figure 1: Inequalities in Sedentary Time by 10 SEP measures for three Scottish cohorts

Legend: The inequalities represent SII (with 95% confidence intervals) for each of the following SEP measures (unless otherwise stated in parentheses) the Scottish Index of Multiple Deprivation, Carstairs deprivation score, Lifetime social class, Parental social class, Equivalised household income, Subjective social position, Highest educational qualification, School leaving age (- stayed on beyond minimum reference) Housing tenure (Binary measure –owners reference) and Car ownership (Binary measure – car owners reference).

For peer review only

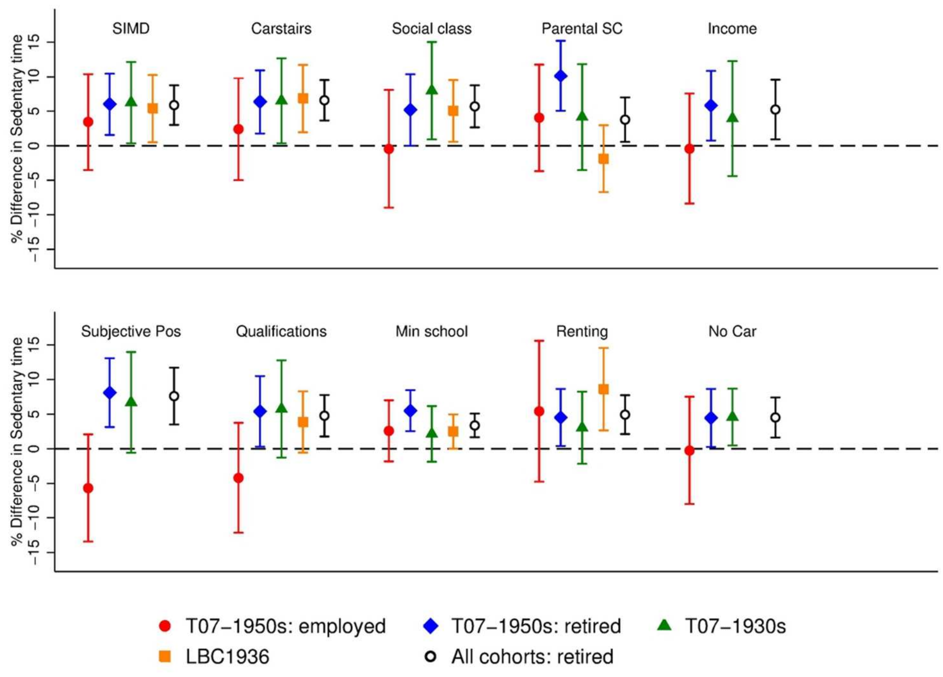

Figure 1: Inequalities in Sedentary Time by 10 SEP measures for three Scottish cohorts. ├ ┬ The inequalities represent SII (with 95% confidence intervals) for each of the following SEP measures (unless otherwise stated in parentheses) the Scottish Index of Multiple Deprivation, Carstairs deprivation score, Lifetime social class, Parental social class, Equivalised household income, Subjective social position, Highest educational qualification, School leaving age (- stayed on beyond minimum reference) Housing tenure (Binary measure –owners reference) and Car ownership (Binary measure – car owners reference). A positive difference (point above the dotted line) indicates that a socioeconomically disadvantaged person is more sedentary a negative difference (point below the line) indicates the reverse.

101x73mm (300 x 300 DPI)

only

APPENDIX

The socioeconomic position (SEP) measures used in this study are as follows:

Lifetime social class was classified using the Registrar General's Socioeconomic Classification RGSC.[1] For LBC1936 this was assessed by asking participants what their main qualification was in wave 1 (2004 to 2007). While for the two Twenty07 cohorts this was assessed by using the highest achieved household social class across all 5 waves of the Twenty07 study between 1987 and 2008.

Parental social class was classified using RGSC for 1951 (professional, managerial, skilled, semiskilled and unskilled)[2] based on retrospective data. For LBC1936 the measure refers to father's social class at age 11 and was collected at wave 1 (2004/2007). For the Twenty07 cohorts the measure is for the head of household's social class at age 15 and was measured at wave 1 (1987/1988).

For the Twenty07 cohorts highest educational qualification (none, standard grades or equivalent, highers or equivalent, HND or equivalent, university degree or equivalent), was collated across all 5 waves of the twenty07 study from 1987 to 2008. For LBC1936 the highest educational qualification (No qualifications, O-level equivalent, A-level or equivalent, Semi-Professional/Professional qualification, Degree) was assessed at wave 1 (2004 to 2007).

Data on age at first leaving full-time education, recorded in 2007/8 for Twenty-07, and school leaving, recorded in 2004 to 2007 for LBC 1936, were used to calculate minimum school leaving age, which was age 14 for the the1930s cohort, and age 15 for the 1950s cohort and the LBC1936.

Twenty07 participants were asked what their net income was after tax, but including benefits and pensions, in 2007/8. These scores were then equivalized to account for household composition using McClements scales.[3]

We used participants' datazone of residence to generate SEP measures contemporaneous to Seniors USP in the form of two area deprivation measures, the Scottish Index of Multiple Deprivation 2012 (SIMD)[4] and Carstairs deprivation score.[5] SIMD is a measure of relative deprivation that includes 7 domains including income, employment, education, health, crime and housing, which are used to create an overall deprivation score for each datazone. The Carstairs deprivation score was calculated using the 2011 census and is the sum of four standardised variables the percentage of people living in household with no access to a car or van, percentage of male unemployment, percentage of private households overcrowded, percentage of households with the household reference person in social class IV or V.[6]

Housing tenure (Own or Mortgage versus Renting or Other) was recorded between 2011/2013 for LBC1936 and 2007/8 for 1950s and 1930s cohorts.

In addition, in 2007/8 data for Twenty07 cohorts, data was collected on car ownership and subjective social status. Car ownership was assessed by asking participants if they or their household owned a car or van. Subjective social status was assessed using a self-anchoring scale in the form of a 10 rung ladder representing society[7] and participants were asked to indicate where they considered themselves to be in relation to others in Britain at the present time.

For peer review only

Appendix table A1: Regression coefficients for the association between SEP measures and sedentary time.

	T07 1950s: Employed		T07 1950s: Retired		T07 1930s: All		LBC1936: All	
	Coef (95% CI)	R^2	Coef (95% CI)	R^2	Coef (95% CI)	R^2	Coef (95% CI)	R^2
**Tenure (Ref = Own/Mortgage)**								
Renting/other	5.42 (-4.76 to 15.59)	0.010	4.52 (0.4 to 8.64)*	0.023	3.04 (-2.16 to 8.24)	0.012	8.61 (2.65 to 14.57)**	0.029
**Car Ownership (Ref = No)**								
Yes	0.26 (-7.51 to 8.03)	0.000	-4.46 (-8.64 to -0.27*)	0.022	-4.57 (-8.68 to -0.47)*	0.041		
**Lifetime Social class (Ref = Professional)**								
II Managerial	1.98 (-3.21 to 7.16)	0.014	1.14 (-2.55 to 4.83)	0.023	6.3 (0.43 to 12.17)*	0.074	2.6 (-0.54 to 5.74)	0.039
III nm Skill non-manual	-1.31 (-8.01 to 5.4)		3.6 (-0.73 to 7.94)		5.76 (-0.51 to 12.03)+		0.39 (-3.34 to 4.12)	
III m Skilled manual	2.7 (-10.93 to 16.33)		5.64 (-1.2 to 12.48)		6.88 (-0.39 to 14.15)+		5.43 (1.37 to 9.49)**	
IV/V Semiskilled / unskilled	0.89 (-21.91 to 23.7)		2.23 (-6.03 to 10.5)		13.46 (4.03 to 22.89)***		6.63 (-0.17 to 13.42)+	
**Highest Qualification (Ref = None)**								
Basic	3.88 (-3.66 to 11.42)	0.012	1.52 (-4.07 to 7.1)	0.034	-5.54 (-10.09 to -0.99)*	0.049	-2.27 (-6.11 to 1.56)	0.008
Professional or Degree	4.4 (-3.56 to 12.37)		-2.42 (-8.04 to 3.2)		-2.69 (-8.36 to 2.98)		-3.02 (-6.98 to 0.94)	
**Minimum School leaving age (Ref = No)**								
Yes	2.59 (-1.82 to 7)	0.013	5.5 (2.54 to 8.46)***	0.064	2.14 (-1.88 to 6.16)	0.010	2.48 (0.01 to 4.96)*	0.014
**Parental social class (Ref = III Skilled)**								
I Professional	1.11 (-8.38 to 10.59)	0.052	-5.11 (-10.41 to 0.18)+	0.098	0.35 (-9.15 to 9.85)	0.039	-0.97 (-5.85 to 3.9)	0.011
II Intermediate Occupations	-4.97 (-10.64 to 0.69)+		-7.43 (-11.39 to -3.48)***		-6.64 (-13.22 to -0.06)*		-0.66 (-3.93 to 2.62)	
IV Partly Skilled manual	-3.86 (-10.29 to 2.57)		2.12 (-1.87 to 6.12)		-0.39 (-6.62 to 5.85)		-2.91 (-7.13 to 1.32)	
V Unskilled	2.72 (-4.31 to 9.76)		-0.17 (-5.46 to 5.12)		-0.74 (-7.75 to 6.27)		-3.14 (-8.68 to 2.4)	
**SIMD**								
Slope Index of Inequality	3.44 (-3.52 to 10.4)	0.009	6.01 (1.56 to 10.46)**	0.035	6.25 (0.35 to 12.15)*	0.036	5.39 (0.5 to 10.27)*	0.017
**Carstairs**								
Slope Index of Inequality	2.39 (-4.98 to 9.76)	0.004	6.35 (1.76 to 10.94)**	0.036	6.51 (0.34 to 12.68)*	0.036	6.84 (1.94 to 11.73)**	0.027
**Gender (Ref= Male)**								
Female	-5.59 (-9.86 to -1.31)*	0.059	-0.85 (-3.83 to 2.13)	0.002	0.5 (-3.49 to 4.5)	0.001	-4.69 (-7.11 to -2.26)***	0.051
**Equivilized Household Income**								
Per £100 increase in Net income	0.15 (-0.57 to 0.86)	0.002	-1.74 (-2.73 to -0.75)***	0.064	-0.51 (-1.52 to 0.5)	0.012		
Per £100 increase income squared			0.08 (0.03 to 0.12)***					
**Subjective social position**								
Each rung on Ladder	1.05 (-0.41 to 2.5)	0.019	-1.46 (-2.3 to -0.63)***	0.058	-1.33 (-2.61 to -0.05)*	0.037		

For peer review only

APPENDIX REFERENCES

1 Office of Population Censuses and Surveys. London: HMSO 1980.

2 Registrar General. The Registrar General's Decennial Supplement, England and Wales 1951, Occupational Mortality Part II, Vol. 1. *Commentary HMSO, London* 1958.

3 McClements LD. Equivalence scales for children. *J Public Econ* 1977;**8**:191-210.

4 Scottish Government. Scottish Index of Multiple Deprivation. 2012.

5 Carstairs V, Morris R. *Deprivation: explaining differences in mortality between Scotland and England and Wales* 1989.

6 Brown D, Allik M, Dundas R*, et al.* Carstairs Scores for Scottish Postcode Sectors, Datazones and Output Areas from the 2011 Census. 2014.

7 Singh-Manoux A, Adler NE, Marmot MG. Subjective social status: its determinants and its association with measures of ill-health in the Whitehall II study. *Soc Sci Med* 2003;**56**:1321-33.

For peer review only

STROBE Statement—Checklist of items that should be included in reports of ***cohort studies***

	Item No	Recommendation
**Title and abstract**	1	(*a*) Indicate the study's design with a commonly used term in the title or the abstract<br>*The term "Prospective cohort" is used in the title.*
		(*b*) Provide in the abstract an informative and balanced summary of what was done and what was found<br>*See abstract on page 2.*
**Introduction**		
Background/rationale	2	Explain the scientific background and rationale for the investigation being reported<br>*The introduction sets out rationale for the study on page 4*
Objectives	3	State specific objectives, including any prespecified hypotheses<br>*Aim of study is set out in last paragraph of introduction on page 4.*
**Methods**		
Study design	4	Present key elements of study design early in the paper<br>*The study design is described on pages 4 to 5.*
Setting	5	Describe the setting, locations, and relevant dates, including periods of recruitment, exposure, follow-up, and data collection<br>*Dates and locations are described in the first three paragraphs of the methods section on pages 4 to 5.*
Participants	6	(*a*) Give the eligibility criteria, and the sources and methods of selection of participants. Describe methods of follow-up<br>*Eligibility criteria described on page 5.*
		(*b*) For matched studies, give matching criteria and number of exposed and unexposed<br>*Not applicable.*
Variables	7	Clearly define all outcomes, exposures, predictors, potential confounders, and effect modifiers. Give diagnostic criteria, if applicable<br>*Variables are summarised on pages 5 to 6 with additional information provided in the supplementary file.*
Data sources/ measurement	8*	For each variable of interest, give sources of data and details of methods of assessment (measurement). Describe comparability of assessment methods if there is more than one group<br>*Summary of sources of data are provided in the main text file on pages 5 to 6 with more extensive details provided in supplementary document.*
Bias	9	Describe any efforts to address potential sources of bias<br>*Test for potential bias due to socioeconomic position are discussed on pages 6 and 8.*
Study size	10	Explain how the study size was arrived at<br>*This is covered on page 5 of the methods section.*
Quantitative variables	11	Explain how quantitative variables were handled in the analyses. If applicable, describe which groupings were chosen and why<br>*The variables areas described in the methods section on pages 5 to 6 and groupings discussed in the results section on page 6.*
Statistical methods	12	(*a*) Describe all statistical methods, including those used to control for confounding<br>*Statistical methods are described on page 6.*
		(*b*) Describe any methods used to examine subgroups and interactions<br>*See statistical methods section on page 6.*

For peer review only

For peer review only - http://bmjopen.bmj.com/site/about/guidelines.xhtml

		(*c*) Explain how missing data were addressed<br>*We present possible evidence of some biases on page 6 and 8.*
		(*d*) If applicable, explain how loss to follow-up was addressed<br>*We tested for potential biased on page 6 and 8.*
		(*e*) Describe any sensitivity analyses<br>*Results from alternative ways of coding independent variables are presented in supplementary file.*
**Results**		
Participants	13*	(a) Report numbers of individuals at each stage of study—eg numbers potentially eligible, examined for eligibility, confirmed eligible, included in the study, completing follow-up, and analysed<br>*This is covered on page 6 of the results .*
		(b) Give reasons for non-participation at each stage
		(c) Consider use of a flow diagram
Descriptive data	14*	(a) Give characteristics of study participants (eg demographic, clinical, social) and information on exposures and potential confounders<br>*This is presented in table 1 on page 7.*
		(b) Indicate number of participants with missing data for each variable of interest<br>*This is presented on table 1 on page 7.*
		(c) Summarise follow-up time (eg, average and total amount)<br>*Study carried out in discrete waves so not applicable.*
Outcome data	15*	Report numbers of outcome events or summary measures over time<br>*Variations in outcome are described in table 2 on page 9.*
Main results	16	(*a*) Give unadjusted estimates and, if applicable, confounder-adjusted estimates and their precision (eg, 95% confidence interval). Make clear which confounders were adjusted for and why they were included<br>*Confidence intervals are presented in the results on page 8 and in figure 1on page 16.*
		(*b*) Report category boundaries when continuous variables were categorized<br>*Not applicable.*
		(*c*) If relevant, consider translating estimates of relative risk into absolute risk for a meaningful time period<br>*Not applicable.*
Other analyses	17	Report other analyses done—eg analyses of subgroups and interactions, and sensitivity analyses<br>*Additional analyses presented as a supplementary file.*
**Discussion**		
Key results	18	Summarise key results with reference to study objectives<br>*Summary presented at start of discussion page 10.*
Limitations	19	Discuss limitations of the study, taking into account sources of potential bias or imprecision. Discuss both direction and magnitude of any potential bias<br>*Limitations discussed on page 11.*
Interpretation	20	Give a cautious overall interpretation of results considering objectives, limitations, multiplicity of analyses, results from similar studies, and other relevant evidence<br>*Discussed on pages 10 to 11.*
Generalisability	21	Discuss the generalisability (external validity) of the study results

For peer review only

For peer review only - http://bmjopen.bmj.com/site/about/guidelines.xhtml

		Generalizability is discussed on page 11.
**Other information**		
Funding	22	Give the source of funding and the role of the funders for the present study and, if applicable, for the original study on which the present article is based<br>*Funding included in the acknowledgements on page 12.*

*Give information separately for exposed and unexposed groups.

Note: An Explanation and Elaboration article discusses each checklist item and gives methodological background and published examples of transparent reporting. The STROBE checklist is best used in conjunction with this article (freely available on the Web sites of PLoS Medicine at http://www.plosmedicine.org/, Annals of Internal Medicine at http://www.annals.org/, and Epidemiology at http://www.epidem.com/). Information on the STROBE Initiative is available at http://www.strobe-statement.org.

For peer review only

For peer review only - http://bmjopen.bmj.com/site/about/guidelines.xhtml

BMJ Open

Relationships between socioeconomic position and objectively measured sedentary behaviour in older adults in three prospective cohorts

Journal:	*BMJ Open*
Manuscript ID	bmjopen-2017-016436.R1
Article Type:	Research
Date Submitted by the Author:	03-May-2017
Complete List of Authors:	Shaw, Richard; University of Glasgow, MRC/CSO Social and Public Health Sciences Unit<br>Čukić, Iva ; University of Edinburgh, Centre for Cognitive Ageing and Cognitive Epidemiology<br>Deary, Ian; University of Edinburgh, Centre for Cognitive Ageing and Cognitive Epidemiology<br>Gale, Catharine; University of Southampton, MRC Lifecourse Epidemiology Unit; University of Edinburgh, Centre for Cognitive Ageing and Cognitive Epidemiology<br>Chastin, Sebastian; Glasgow Caledonian University, School of Health and Life Sciences; Universiteit Gent, Department of Movement and Sports Sciences<br>Dall, Philippa; Glasgow Caledonian University, Institute for applied Health Research, School of Health and Life Sciences<br>Skelton, Dawn; Glasgow Caledonian University, Institute for Applied Health Research, School of Health and Life Sciences<br>Der, Geoff; University of Glasgow, MRC/CSO Social & Public Health Sciences Unit
**Primary Subject Heading**:	Public health
Secondary Subject Heading:	Sociology, Epidemiology
Keywords:	EPIDEMIOLOGY, PUBLIC HEALTH, SOCIAL MEDICINE, SPORTS MEDICINE

SCHOLARONE™
Manuscripts

Relationships between socioeconomic position and objectively measured sedentary behaviour in older adults in three prospective cohorts

Richard J. Shaw[1], Iva Čukić[2], Ian J. Deary[2], Catharine R. Gale[2,3], Sebastien F. M. Chastin[4,5], Philippa M. Dall[4], Dawn A. Skelton[4], Geoff Der[1,2] on behalf of the Seniors USP Team

Affiliations

1 MRC/CSO Social and Public Health Sciences Unit, University of Glasgow, Glasgow, United Kingdom.

2 Department of Psychology Centre for Cognitive Ageing & Cognitive Epidemiology, University of Edinburgh, Edinburgh, UK.

3 MRC Lifecourse Epidemiology Unit, University of Southampton, Southampton, UK.

4 Institute for Applied health Research, School of Health and Life Sciences, Glasgow Caledonian University, Glasgow, UK.

5 Department of Movement and Sports Sciences, Faculty of Medicine and Health Science, Ghent University, Ghent, Belgium.

Word count: 3870

Corresponding Author: Richard Shaw
Address: MRC/CSO Social and Public Health Sciences Unit, University of Glasgow,
200 Renfield street, Glasgow, G2 3QB, UK.
Email: richard.shaw@glasgow.ac.uk
Telephone: + 44 (0) 141 353 7565
Fax: + 44 141 337 2389

For peer review only - http://bmjopen.bmj.com/site/about/guidelines.xhtml

ABSTRACT

Objectives: To Investigate whether sedentary behaviour in older adults is associated with a systematic and comprehensive range of socioeconomic position (SEP) measures across the lifecourse. SEP measures included prospective measures of social class, income, educational qualifications and parental social class and contemporaneous measures of area deprivation.

Setting: Glasgow and the surrounding (West of Scotland) combined with Edinburgh and the surrounding area (the Lothians).

Participants: Community dwelling adults aged around 79, 83, and 64 years from, respectively, the Lothian Birth Cohort 1936 (LBC1936) (n=271) and the 1930s (n=119) and 1950s (n=310) cohorts of the West of Scotland Twenty-07 study

Primary outcome measure: Sedentary behaviour was measured objectively using an activPAL activity monitor worn continuously for seven days, and used to calculate percentage of waking time spent sedentary.

Results: Among retired participants, for most cohort and SEP combinations, greater social disadvantage was associated with increased sedentary time. For example, in the Twenty-07 1930s cohort those most deprived on the Carstairs measure spent 6.5% (95% CI 0.3 to 12.7) more of their waking time sedentary than the least deprived. However, for employed people the relationship between SEP and sedentary behaviour was much weaker For example, in terms of social class differences, among the retired the most disadvantaged spent 5.7% more waking time sedentary (95% CI 2.6% to 87%) whereas among the employed there was effectively no difference (-0.5%; 95% CI -9.0 to 8.0.

Conclusions: Diverse SEP measures were associated with increased sedentary behaviour among retired people. There was little evidence for a relationship between SEP measures and sedentary behaviour among employed older adults. Prior to retirement the constraints of the workplace may be masking effects which are only apparent at weekends.

For peer review only

Strengths and limitations of this study

- Sedentary behaviour is objectively measured using an activPAL monitor that correctly identifies posture.
- Data is available for an entire seven day period thus minimising any systematic variation over the course of the week.
- Our study is the first that we are aware of that uses a comprehensive and systematic range of SEP measures across the lifecourse.
- Due to attrition the sample is more socioeconomically advantaged than the original populations from which it was drawn.

For peer review only

For peer review only - http://bmjopen.bmj.com/site/about/guidelines.xhtml

Sedentary behaviour, defined as energy expenditure ≤ 1.5 metabolic equivalents (METs) while awake and in a sitting or reclining posture,[1] is emerging as a modifiable risk factor for poor health independent of physical activity.[2, 3] There is evidence for associations with: mortality,[3-5] cancer incidence,[3] diabetes,[6] bone density[7] and falls.[8] Sedentary behaviour increases with age.[9] On average, sedentary time represents 65–80 % of an older adult's waking day[10] and 67 % of older adults spend in excess of 8.5 h per day sitting.[11] Reducing sedentary behaviour may, therefore, be an important health message for older adults.

Socio economic position (SEP) has been described as a fundamental cause of poor health and health inequalities.[12] SEP represents flexible resources that shape people's opportunities and provide support for their efforts to engage health enhancing behaviours.[12] As such, it is a multi-dimensional concept[13] with different aspects of socio-economic position being salient for different health outcomes and the importance of those aspects varying across the life course.[14]

The current literature on the socioeconomic determinants of sedentary behaviour in older adults is very limited.[9, 15] Of the few studies that exist most rely on self-reported measures of TV viewing and screen time.[16-22] However, these represent only two of the circumstances in which people might be sedentary. Moreover, self-reported measures of sedentary behaviour have only low to moderate validity[23] and, therefore, attenuate relationships. They are also subject to recall and social desirability bias.

To our knowledge, only five studies have examined the association of SEP with objectively measured sedentary behaviour in older people and these have all used ActiGraph accelerometers worn on the waist,[24-28] which do not accurately record posture.[29] Moreover, these studies did not explicitly focus on SEP as a potential determinant of sedentary behaviour. In short, while results from younger adults suggest SEP could be an important predictor of sedentary behaviour,[30] the situation for older adults remains unclear.

In this study we investigate whether SEP is an important determinant of sedentary behaviour among older people. We use data from three Scottish cohorts aged in their sixties, seventies and eighties. Sedentary behaviour is measured using the activPAL monitor worn continuously for seven days. As the participants are drawn from existing, longstanding cohorts, our study includes a diverse range of prospective indicators that capture many aspects of socio-economic position including parental social class, education, household social class, neighbourhood deprivation, housing tenure, income and subjective social status.

METHODS

Participants

This study, Seniors USP (Understanding Sedentary Patterns), comprises subsamples of the Lothian Birth Cohort 1936 (LBC1936) and the West of Scotland Twenty-07 study (Twenty-07). Full details for these studies are available elsewhere.[31, 32] The Twenty-07 study itself comprises three age cohorts although only the two oldest are included here (hereafter, referred to as the 1930s and 1950s cohorts according to their decade of birth). Data for the main Twenty-07 study were collected

For peer review only - http://bmjopen.bmj.com/site/about/guidelines.xhtml

in five waves of interviews between 1987 and 2008. LBC1936 is an on-going cohort study of older people that began in 2004 as a follow up to the Scottish Mental Survey 1947.

Data specific to this study, including objective sedentary behaviour, were collected between November 2014 and April 2016 when the mean ages of the cohorts were around 64, 79 and 83 years.

To be eligible to take part in Seniors USP, participants had to live in the community and needed sufficient cognitive ability to be able to provide informed consent and also a sufficiently good memory to complete sleep diaries. Beyond these minimum requirements people were not excluded due to physical or mental impairments. Twenty-07 participants were eligible if they lived within the greater Glasgow area. All eligible people in the 1930s cohort were approached and a random sample of eligible people in the 1950s cohort was selected. Consecutive recruits to wave 4 of LBC1936 were invited to join Seniors USP until the target sample size of 300 was achieved.

Participants of the original Twenty-07 study were representative of the communities from which they were drawn.[33] The LBC1936 participants were drawn from Scottish Mental Survey which was a whole population sample, but are advantaged with respect to SEP, though all groups are represented. Due to attrition, participants in Seniors USP show some differences from the cohorts from which they are drawn. For the 1950s cohort the analysed sample were more advantaged with respect to the lifetime and parental social class, subjective social position, educational qualifications, minimum school leaving age, tenure and car ownership. For the 1930s cohort the analysed sample had an older school leaving age and higher income, but there were no significant differences for the other SEP measures. For LBC1936 the analysed sample were more advantaged with respect to tenure, educational qualifications and minimum school leaving age but there were no significant differences in lifetime and parental social class.

Sedentary behaviour

Sedentary behaviour was measured using the activPAL monitor (activPAL3c, PAL Technologies Ltd, Glasgow, UK), a small and light (53x35x7mm; 15g) tri-axial accelerometer, worn attached to the anterior thigh of the dominant leg with a waterproof dressing. The monitor samples acceleration at 20Hz, which is then categorised into time spent in sedentary or upright posture based on thigh inclination. Additionally time spent walking is identified based on thigh acceleration. The monitor provides accurate and reliable measurement of sedentary behaviour.[34, 35] Participants were initially interviewed for basic socio-demographic and health information and were then asked to wear the activPAL continuously for seven days, while going about their usual daily activities, including overnight and during bathing or swimming. Participants also kept a diary reporting the time they fell asleep the previous night and the time they woke up for each day of monitoring. Self-reported wake and sleep times from the diary were used to isolate waking data for analysis. Participants without a full 7 days were excluded, in order to avoid making any assumptions about wear time. There was little evidence to suggest that included and excluded participants differed on any of the SEP measures.

The outcome measure is the percentage of waking time spent sedentary, averaged over the seven days (hereafter, sedentary time).

For peer review only - http://bmjopen.bmj.com/site/about/guidelines.xhtml

Socio-Economic Position

We include prospective measures of SEP based on the three major axes of social stratification: education, occupation and income. In addition there are two measures of area deprivation, and one measure each of tenure, car ownership and subjective social position. Full details of these measures including when they were collected and how they differed between LBC1936 and the Twenty-07 are given in the appendix. A brief description follows.

Occupation based measures are parental social class (Professional/intermediate/skilled/semi-skilled/unskilled) and lifetime social class (Professional/managerial/skilled non-manual/skilled manual/semi-skilled or unskilled).

Education measures are: highest educational qualification (none/basic/degree or professional) and whether or not left school at minimum leaving age.

The income measure is net household income equivalised to adjust for household composition using the McClements scales.[36]

Area deprivation measures include the Scottish Index of Multiple Deprivation 2012 (SIMD)[37] and Carstairs deprivation score.[38] Both measures are based on the datazone of the participant's residence. The SIMD comprises seven domains: income, employment, education, health, crime, housing and access to facilities which are combined to create an overall deprivation score. The Carstairs deprivation score is based on four measures from the 2011 census: car ownership, male unemployment, overcrowding and low social class.[39]

Our measure of Housing tenure contrasts home owners with others and likewise our measure of car ownership.

Subjective social status was assessed using a self-anchoring scale in the form of a 10 rung ladder representing society[40] and participants were asked to indicate where they considered themselves to be in relation to others in Britain.

Statistical Methods

Differences between the cohorts were identified using Chi-square tests for the ordinal and binary measures and Anova for the continuous measures. Associations between SEP and outcome measures were estimated using linear regression.

In order to facilitate comparisons between SEP measures made on different scales of measurement we used the slope index of inequality (SII).[41] This involves rescaling the SEP measures to fractional ranks, that is, ranking them and dividing by the sample size. Where there are ties in the data the mid-rank is assigned. For the area deprivation measures, ranks are available for the whole of Scotland. For other measures, ranking is cohort specific. For highest educational qualifications, all 5 ordinal categories (see appendix) that were available for each cohort were used to derive the SII, while three categories were used for presentation in tables. For all measures, higher ranks are assigned to greater disadvantage and the SII can therefore be interpreted as the difference in outcome between the hypothetically most and least disadvantaged.

Additional analyses based on the original scores are presented in the appendix.

For peer review only - http://bmjopen.bmj.com/site/about/guidelines.xhtml
For peer review only

All analyses were conducted using Stata version 13.1.

RESULTS

Seven hundred and sixty two participants took part: 340, 129 and 304 each from the 1950s cohort, 1930s, and LBC1936, respectively. Of these, 700 (91%) provided seven full days of activPAL and sleep diary data. We analysed only those who had full data so no assumptions about wear time would have to be made.

Previous research[42] has suggested that the social patterning of physical activity differs before and after retirement and preliminary analysis of sedentary behaviour in the 1950s cohort showed a similar pattern. Consequently, we have divided this cohort into those still employed, including the semi-retired, vs those no longer employed. We refer to the latter as 'retired' even though not all would consider themselves formally retired.

Table 1 shows the breakdown of the sample by SEP and demographic measures. There were significant cohort differences in all the SEP measures ($p<.01$), except parental social class, reflecting differences between the areas from which they are drawn and secular changes in the occupational structure. For all SEP measures the 1930s cohort are the most disadvantaged.

Within the 1950s cohort there were few differences in SEP measures between the employed and retired. The only significant differences were for gender (0.001), only 36.4% of 1950s employed were female compared to 62.5% of the retired, and housing tenure for which 14.0% of the retired were renting compared to only 4.6% of the employed.

Table 2 shows the mean and standard deviation of sedentary time by cohort. As might be expected given their ages, the 1930s cohort are the most sedentary, on average spending 68.2% of the day sedentary. Among the 1950s cohort the retired have similar levels to LBC1936 (62.2% and 62.5%, respectively) whereas the employed have the lowest level (58.3%). Despite LBC1936 being closer in age to the 1930s cohort, their sedentary time is more like the 1950s retired. As the 1930s cohort is the most deprived and LBC1936 the least, it may be that the overall cohort differences reflect the differences in SEP as well as age differences.

Throughout the table there are numerous examples of SEP differences in sedentary time although the small numbers in some categories, especially the extremes of some social class measures, need to be borne in mind.

A comparison of the analysed sample with the remainder of those invited showed a number of differences (data not shown). For LBC1936 there were no significant differences in lifetime and parental social class, gender or self-rated health, but the analysed sample were more advantaged with respect to tenure, educational qualifications and minimum school leaving age. For the 1950s cohort the analysed sample were more advantaged with respect to all the prospective SEP measures and self-rated health but there was no difference in gender. For the 1930s cohort the analysed sample had better self-rated health and older school leaving age, but there were no significant differences for the other SEP measures.

For peer review only - http://bmjopen.bmj.com/site/about/guidelines.xhtml

Table 1: Descriptive statistics by cohort and employment status during Seniors USP

	Twenty-071950s Employed n=110		Twenty-07 1950s Retired n=200		T07 1930s All n=119		LBC 1936 All n=271	
	n	%	n	%	n	%	n	%
**Tenure**								
Own or Mortgage	104	94.6	171	85.5	95	79.8	259	95.6
Renting or Other	5	4.6	28	14.0	21	17.7	12	4.4
Missing	1	0.9	1	0.5	3	2.5	0	0.0
**Car Ownership**								
No	9	8.2	27	13.5	42	35.3		
Yes	100	90.9	172	86.0	74	62.2		
Missing	1	0.9	1	0.5	3	2.5		
**Lifetime Social class RGSC**								
I Professional	27	24.6	45	22.5	18	15.1	69	25.5
II Managerial	60	54.6	94	47.0	47	39.5	101	37.3
III Skilled non manual	19	17.3	43	21.5	31	26.1	50	18.5
III Skilled manual	3	2.7	11	5.5	16	13.5	38	14.0
IV/V Semi-skilled un skilled	1	0.9	7	3.5	7	5.5	10	3.7
Missing	0	0.0	0	0.0	0	0.0	3	1.1
**Highest qualification**								
None	10	9.1	15	7.5	34	28.6	36	13.3
Basic	65	59.1	96	48.0	61	51.3	133	49.1
Degree or Professional qualification	35	31.8	89	44.5	24	20.2	102	37.6
**Minimum School leaving age**								
Stayed past min age	69	62.7	131	65.5	61	51.3	144	53.1
Left minimum or earlier	40	36.4	68	34.0	55	46.2	127	46.9
Missing	1	0.9	1	0.5	3	2.5	0	0
**Parental Social Class**								
I Professional	6	5.5	16	8.0	6	5.0	20	7.4
II Intermediate	21	19.1	33	16.5	14	11.8	54	19.9
III skilled (manual & non-manual)	54	49.1	98	49.0	61	51.3	136	50.2
IV Partly Skilled	15	13.6	32	16.0	16	13.5	28	10.3
V Un skilled	12	10.9	16	8.0	12	10.1	15	5.5
Missing	2	1.8	5	2.5	10	8.4	18	6.6
**Gender**								
Male	70	63.6	75	37.5	54	45.4	140	51.7
Female	40	36.4	125	62.5	65	54.6	131	48.3
**SIMD**								
Mean	0.37		0.42		0.45		0.22	
SD	0.30		0.32		0.33		0.25	
**Carstairs**								
Mean	0.39		0.44		0.47		0.3	
SD	0.29		0.31		0.32		0.25	
**Equivalized Household net Income /£100**								
Mean	5.78		6.22		4.01			
SD	3.09		3.92		2.39			
Missing	11		15		22			
**Subjective social potion**								
Mean	6.52		6.14		6.1			
SD	1.47		1.70		1.62			
Missing	2		3		7			
**Age**								
Mean	64.4		64.7		83.4		79.0	
SD	0.88		0.89		0.62		0.44	

For peer review only - http://bmjopen.bmj.com/site/about/guidelines.xhtml

For peer review only

Table 2: Mean and standard deviation for and percent waking time spent sedentary

	Twenty07 1950s Employed		Twenty07 1950s Retired		Twenty07 1930s All		LBC1936 All	
	Mean	SD	Mean	SD	Mean	SD	Mean	SD
**All members**								
Overall	58.3	11.2	62.2	10.3	68.2	10.9	62.5	10.4
**Tenure**								
Own or Mortgage	58.1	11.4	61.5	10.1	67.8	10.8	62.1	10.2
Renting or Other	63.5	6.6	66.1	10.9	70.8	11.3	70.7	11.9
**Car Ownership**								
No	58.1	11.5	66.0	9.7	71.2	10.7		
Yes	58.4	11.2	61.6	10.3	66.7	10.8		
**Lifetime social class**								
I Professional	57.4	13.3	60.5	9.7	62.5	11.2	60.3	10.1
II Managerial	59.5	10.2	61.6	10.4	68.8	11.5	62.9	10.0
III Skilled non manual	56.1	11.2	64.1	9.7	68.3	9.4	60.7	10.5
III Skilled manual	60.1	14.7	66.2	12.6	69.4	10.0	65.7	10.8
IV/V Semi-skilled un skilled	58.3	-	66.7	13.0	76.0	10.7	66.9	8.9
**Highest achieved qualification**								
None	54.7	13.9	62.6	11.4	71.6	10.0	64.8	11.7
Basic	58.5	11.0	64.1	9.9	66.1	11.4	62.5	10.2
Degree/Prof qualification	59.1	10.8	60.1	10.4	68.9	10.2	61.7	10.1
**Minimum School leaving age**								
Stayed pass min age	57.4	11.4	60.3	9.8	67.3	10.2	61.3	10.1
Left minimum or earlier	60.0	10.8	65.8	10.5	69.4	11.6	63.8	10.5
**Parental Social Class**								
I Professional	60.7	11.8	58.4	9.9	69.7	7.7	66.5	7.2
II Intermediate	54.6	13.1	56.1	9.8	62.7	12.3	62.2	9.4
III skilled (manual & non-manual)	59.6	10.1	63.5	10.2	69.4	5.0	62.5	9.6
IV Partly Skilled	55.7	11.5	65.6	10.0	69.0	14.1	63.2	10.4
V Un skilled	62.3	11.0	63.3	8.8	68.6	15.8	60.3	11.8
**Gender**								
Male	60.4	10.9	62.7	9.1	68.0	12.0	64.8	9.8
Female	54.8	10.8	61.9	11.0	68.5	10.0	60.1	10.5
**SIMD Overall**								
<50 Percentile	57.4	11.4	60.4	9.6	67.0	10.6	61.9	10.2
> 50 Percentile	59.4	10.9	63.9	10.7	69.4	11.2	63.1	10.5
**Carstairs Score**								
< 50 Percentile	57.7	11.8	60.4	9.5	66.8	11.1	60.9	9.6
> 50 Percentile	59.1	10.4	63.8	10.8	69.6	11.6	64.2	10.9
**Income**								
> 50 Percentile	57.6	11.5	60.7	10.0	67.2	11.8		
< 50 Percentile	59.0	10.6	64.0	10.6	71.1	10.4		
**Subjective social position**								
> 50 Percentile	59.5	11.0	60.2	9.3	66.4	10.6		
< 50 Percentile	57.2	11.5	64.5	11.0	69.3	11.4		

For peer review only

Analysis of Sedentary time

Figure 1 presents the results of linear regression analyses regressing sedentary time on the SEP measures. Separate results are shown for: the LBC1936 cohort; the 1930s cohort; the employed and retired subgroups of the 1950s cohort; and the 1950s retired group combined with the two older cohorts. In each case, regression coefficients and their 95% confidence intervals are shown. For the binary SEP measures the effect is simply the difference in sedentary time between the two groups. For the other SEP measures the effect is the SII and is interpretable as the difference in sedentary time between the most and least deprived.

The overall pattern is one of more disadvantaged SEP being associated with greater sedentary time. The exceptions are the employed group and parental social class where there are no clear or consistent patterns. In terms of statistical significance, when the retired people in the 1950s cohort was combined with the two older cohorts all SEP measures were significantly and positively related to sedentary time, the largest SIIs being found for subjective social position (7.6% CI 3.5 to 11.7) and Carstairs deprivation (6.6 % CI 3.6 to 9.5). When these cohorts were investigated separately, relationships between sedentary time and SEP were found for most cohort and SEP combinations. In some cases, particularly for the 1930s cohort, the confidence intervals crossed zero although effects were in the same direction and of similar magnitude. There is limited evidence that the effects of SEP varied by cohort. There was only one significant interaction between an SEP measure and cohort for the retired cohorts which was for parental social class (p=0 .0043).

In contrast to the retired cohorts, none of the SEP measures were significantly associated with sedentary time for the employed in the 1950s cohort. Furthermore half of the coefficients were below zero suggesting that there was no evidence of a relationship between sedentary time and SEP measures in general.

Sensitivity tests and additional analyses

Additional analyses are presented in the appendix. Table A1 includes the regression coefficients for the SEP measures in their original form as shown in Tables 1 and 2. Broadly these are consistent with the results produce by the SII; however, far fewer measures are significant, partly reflecting the reduced statistical power of categorical variables. It should also be noted that there was a non-linear relationship between income and sedentary time among the retired subgroup of the 1950s cohort. This indicates that sedentary time falls with increases in net equivalized household incomes up to about £1,000 a month beyond which further income makes little difference.

Appendix table A2 shows p values for tests of differences between weekdays and weekends in the association between SEP measures and sedentary time. The results for weekdays are presented in appendix figure A1 and weekends in figure A2. We find little evidence of substantive or systematic differences between weekdays and weekends for retired people. For employed people in the 1950s cohort, the majority of SEP measures showed a different association with sedentary behaviour on weekdays from that on weekends. The pattern was one whereby the employed were more similar to the retired at weekends than during weekdays. Some measures, highest qualification, and subjective social position may be associated with reduced sedentary behaviour during the week and show no association at weekends. While SIMD deprivation and parental social class were associated with increased sedentary behaviour at weekends but not during the week. Also in the appendix, figure

For peer review only - http://bmjopen.bmj.com/site/about/guidelines.xhtml

A3, shows inequalities in step count using the same methodology shown for sedentary time in figure 1. The results for step count mirror those for sedentary time albeit with slightly weaker relationships.

DISCUSSION

Among older, retired adults we find consistent evidence that socially disadvantaged people with respect to nearly all SEP measures are more sedentary than their advantaged counterparts. In contrast there was little evidence of a consistent relationship between SEP and sedentary time averaged over the week for those still employed.

We identified only five previous studies that measured sedentary behaviour objectively in older adults and included measures of SEP.[24-28] All of these used ActiGraph accelerometers worn on the hip which are unable to consistently distinguish sitting from standing.[29] Van Holle et al. included measures of education, occupation and income but did not examine their association with sedentary behaviour.[25] The other three studies present a mixed picture. Of the three that included a measure of education, higher educational attainment was significantly associated with lower sedentary time in two [27, 28] whilst the more educated were more sedentary in the other,[26] although the significance of the latter was not clear. Three studies included occupation, grade or social class[24, 27, 28] but there was no significant association with sedentary behaviour in any of them. One study included income which was not significantly associated with sedentary behaviour.[26]

Aside from the different measurement of sedentary behaviour there are possible explanations for these differences. In the study by Dunlop et al.[26] 47% of the NHANES sample were aged 60-69 so a large proportion would still be employed and, if the pattern observed here is true of the USA, this might obscure the relationship amongst the retired. In the study by van der Berg et al.[27] the occupational classification was relatively crude with little discrimination amongst the women, 69% of whom were homemakers. Sartini et al.[24] used a dichotomised social class as a covariate in mutually adjusted models containing a large number of variables several of which might plausibly lie on the causal pathway.

Investigations of the influence of neighbourhood SEP among older adults is limited to studies of self-reported sedentary behaviour in which neighbourhoods have been dichotomised into low and high income.[17, 21] We are not aware of any studies investigating the influence of housing tenure, car ownership or subjective social position on sedentary time.

The most important result of our study is that social inequalities in sedentary behaviour averaged over the week are greater in retired people than among the employed. This may be partly due to how employment constrains people's behaviour. Studies of working age adults such as Van Dyck et al (2010),[30] suggest that white collar workers may in fact be more sedentary. Other research has shown that on retirement manual workers lose activity gained from work which is not compensated for by increasing leisure time activity.[42] Given that our study was designed to investigate sedentary behaviour in older people, we only have a small sample of employed people and this should be investigated further in a working age cohort.

For peer review only - http://bmjopen.bmj.com/site/about/guidelines.xhtml

We find relationships even with measures set relatively early in life such as school leaving age, suggesting that the pathways between SEP and sedentary behaviour may be established early in life.

Evidence on why socioeconomically disadvantaged people are more likely to be sedentary in retirement is sparse and explanations are likely to be a complex interplay of social, physical, cultural environments and health.[9, 43] In addition, while it is relatively well established within the physical activity literature that socioeconomically disadvantaged people are less active, there has been little exploration of the pathways.[44] Qualitative research indicates health is a key determinant of sedentary behaviour,[45] and there is evidence that obesity may cause sedentary behaviour[46] rather than necessarily be a consequence. However, for these cohorts health and obesity are more likely to mediate the relationship between SEP and sedentary behaviour than be a confounder. The obesogenic environment which had led to socio-inequalities in obesity was not well established until these cohorts were in midlife or later.[47] This is after most lifecourse critical periods of social mobility during which obesity could determine socioeconomic position.

One possible explanation for these results is that workers in more physically-active occupations are less likely to develop active leisure activities during working life and this carries on into retirement.[42] Additionally a qualitative study has indicated that financial costs may prevent people taking part in activities that encourage people to be active.[48]

Strengths and limitations

Our study has the most comprehensive range of SEP measures in any investigation of sedentary behaviour that we are aware of. The measures are made at the individual, household and area level and pertain to different stages throughout the lifecourse, some quite distal to the outcomes. The area level measures are obtained by geocoding the participant's residential address and so are objective. Even those that were self-reported were ascertained prior to, and independently of, the measurement of sedentary behaviour. Finding consistent results across such a wide range of measures suggests that our results are unlikely to be due to chance.

We used the activPAL3 monitor which provides an objective measure of sedentary behaviour that correctly identifies posture. The activPAL is also worn continuously whereas hip worn monitors are typically removed at night, and when showering or bathing, which introduces additional sources of error.

The data cover an entire seven day period thus minimising any systematic variation over the course of the week. A full week of data was available for a very high proportion of the participants.

We used the slope index of inequality to facilitate comparison of SEP indicators made on different scales.

Sampling from existing cohorts also has its drawbacks. As longitudinal studies progress they increasingly suffer from attrition and survival bias. Furthermore, the sample included here is generally more advantaged than the cohorts from which they were drawn. However, it is unclear whether this will have biased the relationships estimated here. In this paper we have focused on an overall measure of sedentary behaviour. It is possible that information on the context in which sedentary behaviour occurs would provide the detail needed to explain the patterns observed here.[49]

For peer review only - http://bmjopen.bmj.com/site/about/guidelines.xhtml

Another weakness of our approach is the reliance on self-reports of sleep and waking times. Efforts to accurately identify sleep time from accelerometry data might prove fruitful for future research.

CONCLUSIONS

In conclusion, sedentary behaviour appears to be socially patterned among older people after retirement but not before. Prior to retirement the constraints of the workplace may be masking effects which are only apparent at weekends. The results here reinforce the message that retirement is a key transition and an opportunity for interventions to improve health and lessen health inequalities. Policies to address health inequalities in later life should provide opportunities and support for older people to develop habits and leisure time activities that replace sedentary behaviour.

For peer review only

For peer review only - http://bmjopen.bmj.com/site/about/guidelines.xhtml

ACKNOWLEDGEMENTS

The named authors present the study on behalf of the Seniors USP Team, which comprises: Dawn A Skelton (PI), Sebastien Chastin, Simon Cox, Elaine Coulter, Iva Čukić, Philippa Dall, Ian Deary, Geoff Der, Manon Dontje, Claire Fitzsimons, Catherine Gale, Jason Gill, Malcolm Granat, Cindy Gray, Carolyn Greig, Elaine Hindle, Karen Laird, Gillian Mead, Nanette Mutrie, Victoria Palmer, Ratko Radakovic, Naveed Sattar, Richard Shaw, John Starr, Sally Stewart, Sally Wyke.

The Lothian Birth Cohort 1936 (LBC1936) thank the cohort members, investigators, research associates and team members. We also thank the radiographers at the Brain Research Imaging Centre, and the research nurses and Genetics Core staff at the Wellcome Trust Clinical Research Facility.

The Twenty -07 study thanks all of the cohort participants, and the survey staff and research nurses who carried out the study. The data are employed here with the permission of the Twenty-07 Steering Committee.

CONTRIBUTORS

SFMC, PMD, IJD, GD, CRG and DAS contributed to the design of the study. GD conceived original idea for the paper and provided statistical support. RJS conducted the analyses and wrote the first draft of the manuscript. IC assisted with preparation of LBC1936 data. IJD is director of the LBC1936 study. CRG provided statistical advice. IC, IJD, CRG drafted LBC1936 methodology. PMD and SFMC contributed to acquisition, analysis and processing of activPAL data. DAS was the principle investigator for Seniors USP and provided gerontological advice. All authors have read and commented on the manuscript and approved the final version.

FUNDING

The Seniors USP (understanding sedentary patterns) project is funded by the UK Medical Research Council (MRC) as part of the Lifelong Health and Wellbeing Initiative (LLHW) [MR/K025023/1]. The West of Scotland Twenty-07 Study was funded by the MRC and the data were originally collected by the MRC Social and Public Health Sciences Unit (MC_A540_53462). LBC1936 data collection are supported by the Disconnected Mind project (funded by Age UK and MRC [Mr/M01311/1 and G1001245/96077]) and undertaken within the University of Edinburgh Centre for Cognitive Ageing and Cognitive Epidemiology (funded by the BBSRC and MRC as part of the LLHW [MR/K026992/1]).

COMPETING INTERESTS

ID is supported by Age UK. PD has received grant funding from PAL technologies outside the submitted work.

ETHICS

Ethics approval for the Twenty-07 West of Scotland study participants was gained for each wave from the NHS and / or Glasgow University Ethics Committees. Ethical approval for LBC1936 was obtained from the Multi-Centre Research Ethics Committee and from Lothian Research Ethics Committee. All participants gave written, informed consent.

For peer review only - http://bmjopen.bmj.com/site/about/guidelines.xhtml

For peer review only

DATA SHARING AGREEMENT

Data collected as part of the Seniors USP study is embargoed until October 2018. Thereafter data sharing will be governed by the agreements already in place for the Twenty-07 and LBC1936 studies.

The West of Scotland Twenty-07 study is managed by the MRC/CSO Social and Public Health Sciences Unit, University of Glasgow. Further information about how to access the data can be found at http://2007study.sphsu.mrc.ac.uk/

Data are available upon request from the Lothian Birth Cohort 1936 Study. To request the data readers should contact the principal investigator, Ian Deary, who can be contacted at i.deary@ed.ac.uk.

For peer review only

For peer review only - http://bmjopen.bmj.com/site/about/guidelines.xhtml

REFERENCES

1 Sedentary Behaviour Research Network. Letter to the Editor: Standardized use of the terms “sedentary” and “sedentary behaviours”. *Appl Physiol Nutr Metab* 2012;**37**:540-2.

2 Owen N, Sugiyama T, Eakin EE*, et al.* Adults' Sedentary Behavior: Determinants and Interventions. *Am J Prev Med* 2011;**41**:189-96.

3 Biswas A, Oh PI, Faulkner GE*, et al.* Sedentary time and its association with risk for disease incidence, mortality, and hospitalization in adults: A systematic review and meta-analysis. *Ann Intern Med* 2015;**162**:123-32.

4 Katzmarzyk PT, Church TS, Craig CL*, et al.* Sitting time and mortality from all causes, cardiovascular disease, and cancer. *Med Sci Sports Exerc* 2009;**41**:998-1005.

5 Hagger-Johnson G, Gow AJ, Burley V*, et al.* Sitting Time, Fidgeting, and All-Cause Mortality in the UK Women’s Cohort Study. *Am J Prev Med* 2016;**50**:154-60.

6 Wilmot EG, Edwardson CL, Achana FA*, et al.* Sedentary time in adults and the association with diabetes, cardiovascular disease and death: systematic review and meta-analysis. *Diabetologia* 2012;**55**:2895-905.

7 Chastin SFM, Mandrichenko O, Helbostadt JL*, et al.* Associations between objectively-measured sedentary behaviour and physical activity with bone mineral density in adults and older adults, the NHANES study. *Bone* 2014;**64**:254-62.

8 Jefferis BJ, Merom D, Sartini C*, et al.* Physical Activity and Falls in Older Men: The Critical Role of Mobility Limitations. *Med Sci Sports Exerc* 2015;**47**:2119-28.

9 Chastin S, Buck C, Freiberger E*, et al.* Systematic literature review of determinants of sedentary behaviour in older adults: a DEDIPAC study. *Int J Behav Nutr Phys Act* 2015;**12**:127.

10 Harvey JA, Chastin SFM, Skelton DA. How Sedentary Are Older People? A Systematic Review of the Amount of Sedentary Behavior. *J Aging Phys Act* 2015;**23**:471-87.

11 Harvey J, Chastin S, Skelton D. Prevalence of Sedentary Behavior in Older Adults: A Systematic Review. *Int J Environ Res Public Health* 2013;**10**:6645-61.

12 Phelan JC, Link BG, Tehranifar P. Social Conditions as Fundamental Causes of Health Inequalities: Theory, Evidence, and Policy Implications. *J Health Soc Behav* 2010;**51**:S28-S40.

13 Krieger N, Williams DR, Moss NE. Measuring Social Class in US Public Health Research: Concepts, Methodologies, and Guidelines. *Annu Rev Public Health* 1997;**18**:341-78.

14 Galobardes B, Shaw M, Lawlor DA*, et al.* Indicators of socioeconomic position (part 1). *J Epidemiol Community Health* 2006;**60**:7-12.

15 Brug J, Chinapaw M. Determinants of engaging in sedentary behavior across the lifespan; lessons learned from two systematic reviews conducted within DEDIPAC. *Int J Behav Nutr Phys Act* 2015;**12**:134.

16 Van Cauwenberg J, De Donder L, Clarys P*, et al.* Relationships of individual, social, and physical environmental factors with older adults' television viewing time. *J Aging Phys Act* 2014;**22**:508-17.

17 Ding D, Sugiyama T, Winkler E*, et al.* Correlates of Change in Adults' Television Viewing Time: A Four-Year Follow-up Study. *Med Sci Sports Exerc* 2012;**44**:1287-92.

18 Smith L, Gardner B, Hamer M. Childhood correlates of adult TV viewing time: a 32-year follow-up of the 1970 British Cohort Study. *J Epidemiol Community Health* 2015;**69**:309-13.

19 Gardner B, Iliffe S, Fox K*, et al.* Sociodemographic, behavioural and health factors associated with changes in older adults' TV viewing over 2years. *Int J Behav Nutr Phys Act* 2014;**11**:102.

20 Busschaert C, Scherrens A-L, De Bourdeaudhuij I*, et al.* Socio-Ecological Variables Associated with Context-Specific Sitting Time in Belgian Older Adults: A One-Year Follow-Up Study. *PLOS ONE* 2016;**11**:e0167881.

21 Sugiyama T, Salmon J, Dunstan DW*, et al.* Neighborhood Walkability and TV Viewing Time Among Australian Adults. *Am J Prev Med* 2007;**33**:444-9.

22 Clark BK, Sugiyama T, Healy GN*, et al.* Socio-demographic correlates of prolonged television viewing time in Australian men and women: the AusDiab study. *J Phys Act Health* 2010;**7**:595-601.

For peer review only - http://bmjopen.bmj.com/site/about/guidelines.xhtml

23 Celis-Morales CA, Perez-Bravo F, Ibañez L*, et al.* Objective vs. Self-Reported Physical Activity and Sedentary Time: Effects of Measurement Method on Relationships with Risk Biomarkers. *PLOS ONE* 2012;**7**:e36345.
24 Sartini C, Wannamethee S, Iliffe S*, et al.* Diurnal patterns of objectively measured physical activity and sedentary behaviour in older men. *BMC Public Health* 2015;**15**:609.
25 Van Holle V, Van Cauwenberg J, De Bourdeaudhuij I*, et al.* Interactions between Neighborhood Social Environment and Walkability to Explain Belgian Older Adults' Physical Activity and Sedentary Time. *Int J Environ Res Public Health* 2016;**13**:569.
26 Dunlop DD, Song J, Arntson EK*, et al.* Sedentary time in US older adults associated with disability in activities of daily living independent of physical activity. *J Phys Act Health* 2015;**12**:93.
27 van der Berg JD, Bosma H, Caserotti P*, et al.* Midlife determinants associated with sedentary behavior in old age. *Med Sci Sports Exerc* 2014;**46**:1359-65.
28 Hamer M, Kivimaki M, Steptoe A. Longitudinal patterns in physical activity and sedentary behaviour from mid-life to early old age: a substudy of the Whitehall II cohort. *J Epidemiol Community Health* 2012;**66**:1110-5.
29 Crouter SE, Clowers KG, Bassett DR. A novel method for using accelerometer data to predict energy expenditure. *J Appl Physiol* 2006;**100**:1324-31.
30 Van Dyck D, Cardon G, Deforche B*, et al.* Neighborhood Walkability and Sedentary Time in Belgian Adults. *Am J Prev Med* 2010;**39**:25-32.
31 Deary IJ, Gow AJ, Pattie A*, et al.* Cohort Profile: The Lothian Birth Cohorts of 1921 and 1936. *Int J Epidemiol* 2012;**41**:1576-84.
32 Benzeval M, Der G, Ellaway A*, et al.* Cohort Profile: West of Scotland Twenty-07 Study: Health in the Community. *Int J Epidemiol* 2009;**38**:1215-23.
33 Der G. A comparison of the West of Scotland Twenty-07 study sample and the 1991 Census SARs. . *Working Paper* Glasgow: MRC Medical Sociology Unit 1998.
34 Kozey-Keadle S, Libertine A, Lyden K*, et al.* Validation of wearable monitors for assessing sedentary behavior. *Med Sci Sports Exerc* 2011;**43**:1561-7.
35 Sellers C, Dall P, Grant M*, et al.* Validity and reliability of the activPAL3 for measuring posture and stepping in adults and young people. *Gait Posture* 2016;**43**:42-7.
36 McClements LD. Equivalence scales for children. *J Public Econ* 1977;**8**:191-210.
37 Scottish Government. Scottish Index of Multiple Deprivation. 2012.
38 Carstairs V, Morris R. Deprivation: explaining differences in mortality between Scotland and England and Wales. *BMJ* 1989;**299**:886-9.
39 Brown D, Allik M, Dundas R*, et al.* Carstairs Scores for Scottish Postcode Sectors, Datazones and Output Areas from the 2011 Census. MRC/CSO SPHSU, Glasgow. 2014.
40 Singh-Manoux A, Adler NE, Marmot MG. Subjective social status: its determinants and its association with measures of ill-health in the Whitehall II study. *Soc Sci Med* 2003;**56**:1321-33.
41 Wagstaff A, Paci P, van Doorslaer E. On the measurement of inequalities in health. *Soc Sci Med* 1991;**33**:545-57.
42 Berger U, Der G, Mutrie N*, et al.* The impact of retirement on physical activity. *Ageing Soc* 2005;**25**:181-95.
43 Chastin SFM, De Craemer M, Lien N*, et al.* The SOS-framework (Systems of Sedentary behaviours): an international transdisciplinary consensus framework for the study of determinants, research priorities and policy on sedentary behaviour across the life course: a DEDIPAC-study. *Int J Behav Nutr Phys Act* 2016;**13**:83.
44 Barnett I, van Sluijs EMF, Ogilvie D. Physical Activity and Transitioning to Retirement: A Systematic Review. *Am J Prev Med* 2012;**43**:329-36.
45 Chastin S, Fitzpatrick N, Andrews M*, et al.* Determinants of Sedentary Behavior, Motivation, Barriers and Strategies to Reduce Sitting Time in Older Women: A Qualitative Investigation. *Int J Environ Res Public Health* 2014;**11**:773-91.

For peer review only

For peer review only - http://bmjopen.bmj.com/site/about/guidelines.xhtml

46 Ekelund U, Brage S, Besson H*, et al.* Time spent being sedentary and weight gain in healthy adults: reverse or bidirectional causality? *Am J Clin Nutr* 2008;**88**:612-7.
47 Shaw RJ, Green MJ, Popham F*, et al.* Differences in adiposity trajectories by birth cohort and childhood social class: evidence from cohorts born in the 1930s, 1950s and 1970s in the west of Scotland. *J Epidemiol Community Health* 2014;**68**:550-6.
48 Tam-Seto L, Weir R, Dogra S. Factors influencing sedentary behaviour in older adults: An ecological approach. *Aims Publc Health* 2016;**3**:555-72.
49 Healy GN, Clark BK, Winkler EAH*, et al.* Measurement of Adults' Sedentary Time in Population-Based Studies. *Am J Prev Med* 2011;**41**:216-27.

For peer review only

For peer review only - http://bmjopen.bmj.com/site/about/guidelines.xhtml

Caption Figure 1: Inequalities in Sedentary Time by 10 SEP measures for three Scottish cohorts

Legend: The inequalities represent SII (with 95% confidence intervals) for each of the following SEP measures (unless otherwise stated in parentheses) the Scottish Index of Multiple Deprivation, Carstairs deprivation score, Lifetime social class, Parental social class, Equivalised household income, Subjective social position, Highest educational qualification, School leaving age (- stayed on beyond minimum reference) Housing tenure (Binary measure –owners reference) and Car ownership (Binary measure – car owners reference). A positive difference (point above the dotted line) indicates that a socioeconomically disadvantaged person is more sedentary a negative difference (point below the line) indicates the reverse.

For peer review only

For peer review only - http://bmjopen.bmj.com/site/about/guidelines.xhtml

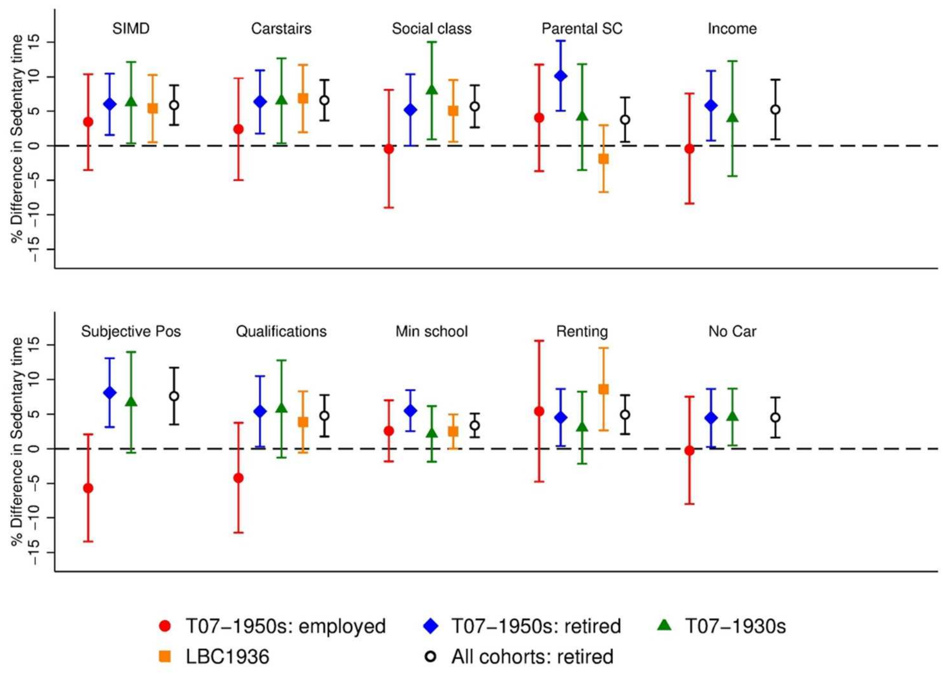

Figure 1: Inequalities in Sedentary Time by 10 SEP measures for three Scottish cohorts. ├ ┬ The inequalities represent SII (with 95% confidence intervals) for each of the following SEP measures (unless otherwise stated in parentheses) the Scottish Index of Multiple Deprivation, Carstairs deprivation score, Lifetime social class, Parental social class, Equivalised household income, Subjective social position, Highest educational qualification, School leaving age (- stayed on beyond minimum reference) Housing tenure (Binary measure –owners reference) and Car ownership (Binary measure – car owners reference). A positive difference (point above the dotted line) indicates that a socioeconomically disadvantaged person is more sedentary a negative difference (point below the line) indicates the reverse.

101x73mm (300 x 300 DPI)

APPENDIX 1: ADDITIONAL DETAILS ON THE COLLECTION AND DERIVATION OF SEP MEASURES

The socioeconomic position (SEP) measures used in this study are as follows:

Lifetime social class was classified using the Registrar General's Socioeconomic Classification RGSC.[1] For LBC1936 this was assessed by asking participants what their main qualification was in wave 1 (2004 to 2007). While for the two Twenty-07 cohorts this was assessed by using the highest achieved household social class across all 5 waves of the Twenty-07 study between 1987 and 2008.

Parental social class was classified using RGSC for 1951 (professional, managerial, skilled, semiskilled and unskilled)[2] based on retrospective data. For LBC1936 the measure refers to father's social class at age 11 and was collected at wave 1 (2004/2007). For the Twenty-07 cohorts the measure is for the head of household's social class at age 15 and was measured at wave 1 (1987/1988).

For the Twenty-07 cohorts highest educational qualification (none, standard grades or equivalent, highers or equivalent, HND or equivalent, university degree or equivalent), was collated across all 5 waves of the twenty-07 study from 1987 to 2008. For LBC1936 the highest educational qualification (No qualifications, O-level equivalent, A-level or equivalent, Semi-Professional/Professional qualification, Degree) was assessed at wave 1 (2004 to 2007).

Data on age at first leaving full-time education, recorded in 2007/8 for Twenty-07, and school leaving, recorded in 2004 to 2007 for LBC 1936, were used to calculate minimum school leaving age, which was age 14 for the the1930s cohort, and age 15 for the 1950s cohort and the LBC1936.

Twenty-07 participants were asked what their net income was after tax, but including benefits and pensions, in 2007/8. These scores were then equivalized to account for household composition using McClements scales.[3]

We used participants' datazone of residence to generate SEP measures contemporaneous to Seniors USP in the form of two area deprivation measures, the Scottish Index of Multiple Deprivation 2012 (SIMD)[4] and Carstairs deprivation score.[5] SIMD is a measure of relative deprivation that includes 7 domains including income, employment, education, health, crime and housing, which are used to create an overall deprivation score for each datazone. The Carstairs deprivation score was calculated using the 2011 census and is the sum of four standardised variables the percentage of people living in household with no access to a car or van, percentage of male unemployment, percentage of private households overcrowded, percentage of households with the household reference person in social class IV or V.[6]

Housing tenure (Own or Mortgage versus Renting or Other) was recorded between 2011/2013 for LBC1936 and 2007/8 for 1950s and 1930s cohorts.

In addition, in 2007/8 data for Twenty-07 cohorts, data was collected on car ownership and subjective social status. Car ownership was assessed by asking participants if they or their household owned a car or van. Subjective social status was assessed using a self-anchoring scale in the form of a 10 rung ladder representing society[7] and participants were asked to indicate where they considered themselves to be in relation to others in Britain at the present time.

For peer review only

Appendix table A1: Regression coefficients for the association between SEP measures and sedentary time.

	T07 1950s: Employed		T07 1950s: Retired		T07 1930s: All		LBC1936: All	
	Coef (95% CI)	R^2	Coef (95% CI)	R^2	Coef (95% CI)	R^2	Coef (95% CI)	R^2
**Tenure (Ref = Own/Mortgage)**								
Renting/other	5.42 (-4.76 to 15.59)	0.010	4.52 (0.4 to 8.64)*	0.023	3.04 (-2.16 to 8.24)	0.012	8.61 (2.65 to 14.57)**	0.029
**Car Ownership (Ref = No)**								
Yes	0.26 (-7.51 to 8.03)	0.000	-4.46 (-8.64 to -0.27*)	0.022	-4.57 (-8.68 to -0.47)*	0.041		
**Lifetime Social class (Ref = Professional)**								
II Managerial	1.98 (-3.21 to 7.16)	0.014	1.14 (-2.55 to 4.83)	0.023	6.3 (0.43 to 12.17)*	0.074	2.6 (-0.54 to 5.74)	0.039
III nm Skill non-manual	-1.31 (-8.01 to 5.4)		3.6 (-0.73 to 7.94)		5.76 (-0.51 to 12.03)+		0.39 (-3.34 to 4.12)	
III m Skilled manual	2.7 (-10.93 to 16.33)		5.64 (-1.2 to 12.48)		6.88 (-0.39 to 14.15)+		5.43 (1.37 to 9.49)**	
IV/V Semiskilled / unskilled	0.89 (-21.91 to 23.7)		2.23 (-6.03 to 10.5)		13.46 (4.03 to 22.89)***		6.63 (-0.17 to 13.42)+	
**Highest Qualification (Ref = None)**								
Basic	3.88 (-3.66 to 11.42)	0.012	1.52 (-4.07 to 7.1)	0.034	-5.54 (-10.09 to -0.99)*	0.049	-2.27 (-6.11 to 1.56)	0.008
Professional or Degree	4.4 (-3.56 to 12.37)		-2.42 (-8.04 to 3.2)		-2.69 (-8.36 to 2.98)		-3.02 (-6.98 to 0.94)	
**Minimum School leaving age (Ref = No)**								
Yes	2.59 (-1.82 to 7)	0.013	5.5 (2.54 to 8.46)***	0.064	2.14 (-1.88 to 6.16)	0.010	2.48 (0.01 to 4.96)*	0.014
**Parental social class (Ref = III Skilled)**								
I Professional	1.11 (-8.38 to 10.59)	0.052	-5.11 (-10.41 to 0.18)+	0.098	0.35 (-9.15 to 9.85)	0.039	-0.97 (-5.85 to 3.9)	0.011
II Intermediate Occupations	-4.97 (-10.64 to 0.69)+		-7.43 (-11.39 to -3.48)***		-6.64 (-13.22 to -0.06)*		-0.66 (-3.93 to 2.62)	
IV Partly Skilled manual	-3.86 (-10.29 to 2.57)		2.12 (-1.87 to 6.12)		-0.39 (-6.62 to 5.85)		-2.91 (-7.13 to 1.32)	
V Unskilled	2.72 (-4.31 to 9.76)		-0.17 (-5.46 to 5.12)		-0.74 (-7.75 to 6.27)		-3.14 (-8.68 to 2.4)	
**SIMD**								
Slope Index of Inequality	3.44 (-3.52 to 10.4)	0.009	6.01 (1.56 to 10.46)**	0.035	6.25 (0.35 to 12.15)*	0.036	5.39 (0.5 to 10.27)*	0.017
**Carstairs**								
Slope Index of Inequality	2.39 (-4.98 to 9.76)	0.004	6.35 (1.76 to 10.94)**	0.036	6.51 (0.34 to 12.68)*	0.036	6.84 (1.94 to 11.73)**	0.027
**Gender (Ref= Male)**								
Female	-5.59 (-9.86 to -1.31)*	0.059	-0.85 (-3.83 to 2.13)	0.002	0.5 (-3.49 to 4.5)	0.001	-4.69 (-7.11 to -2.26)***	0.051
**Equivilized Household Income**								
Per £100 increase in Net income	0.15 (-0.57 to 0.86)	0.002	-1.74 (-2.73 to -0.75)***	0.064	-0.51 (-1.52 to 0.5)	0.012		
Per £100 increase income squared			0.08 (0.03 to 0.12)***					
**Subjective social position**								
Each rung on Ladder	1.05 (-0.41 to 2.5)	0.019	-1.46 (-2.3 to -0.63)***	0.058	-1.33 (-2.61 to -0.05)*	0.037		

For peer review only

APPENDIX 2: DOES THE RELATIONSHIP BETWEEN SEDENTARY TIME AND SOCIOECONOMIC POSTION DIFFER BETWEEN WEEKDAYS AND WEEKENDS RESULTS IN THREE OLDER COHORTS

Methods

The analytic sample is the same as that used in the main paper. However, the analytic approach is different. We used a two level multilevel models with days of the week nested within individuals. Daily percentage waking time spent sedentary is the outcome. The SEP measures, type of day (weekday vs weekend) and the interaction between them are the main predictors. Models also adjust for the order of the day in which people participated. i.e. was it the first, second, … or Seventh day of data collection.

All models were estimated using the xtmixed command in Stata 13.1, and the margins post estimation command was used to obtain the results plotted in Figures A1 and A2. The principal question addressed by the analysis is whether there is a different relationship between SEP and sedentary behaviour on weekdays versus weekends and this is indicates by a significant interaction between the SEP measure and the type of day.

Results

The results of the tests for interaction between SEP measures and type of day are shown in appendix table A2. For the 1950s employed group there are significant interaction for six out of the ten SEP measures: SIMD, lifetime social class, subjective Social position, highest qualification and minimum school leaving age. For the retired groups there were between two and four significant interactions, but there was no great consistency in these across the groups.

The results from these models are shown for weekdays in Appendix Figure A1 and for weekends in Appendix Figure A2. For the retired cohorts there is little to suggest there is much in the way of substantive differences between the relationships for weekdays and weekends. For the 1950s employed group, the results are more similar to the retired groups on weekend days than they are during the week. Indeed, for subjective social position and educational qualifications disadvantage may be associated with reduced sedentary behaviour during the week.

Discussion

Our results would suggest that among retired people the association between SEP and sedentary behaviour does not differ between weekends and weekdays. In contrast for employed people different measures of SEP have different associations with sedentary behaviour on weekdays and weekends. The relative small numbers of employed people that we have in the cohort means that these conclusions should be treated with caution.

For peer review only

Appendix Table A2. p values for interaction terms between weekends and SEP measures in the prediction of Sedentary time.

	1950s Employed	1950s Retired	1930s AI	LBC1936	Retired cohorts combined
SIMD	0.020	0.289	0.537	0.035	0.410
Carstairs Deprivation	0.066	0.210	0.947	0.011	0.471
Lifetime Social class RGSC	0.041	0.207	0.021	0.001	0.605
Parental Social Class	0.006	0.063	0.793	0.114	0.850
Income	0.873	0.514	0.756		0.445
Subjective social position	0.000	0.029	0.815		0.047
Highest qualification	0.019	0.487	0.032	0.280	0.514
Minimum School leaving age	0.002	0.957	0.250	0.254	0.835
Tenure	0.322	0.017	0.025	0.971	0.006
Car Ownership	0.384	0.159	0.048		0.019

For peer review only

Caption Appendix Figure A1. Inequalities in weekday sedentary time for three Scottish cohorts.

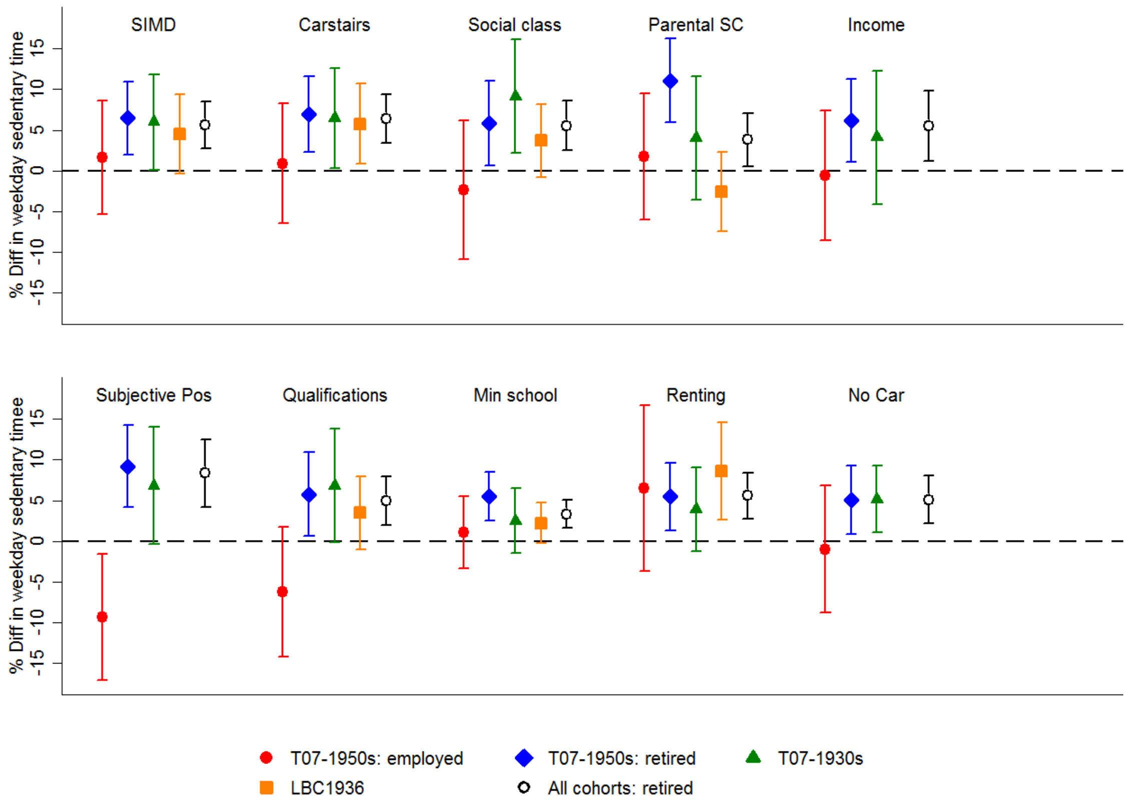

Legend: The inequalities represent SII (with 95% confidence intervals) for each of the following SEP measures (unless otherwise stated in parentheses) the Scottish Index of Multiple Deprivation, Carstairs deprivation score, Lifetime social class, Parental social class, Equivalised household income, Subjective social position, Highest educational qualification, School leaving age (- stayed on beyond minimum reference) Housing tenure (Binary measure –owners reference) and Car ownership (Binary measure – car owners reference). A positive difference (point above the dotted line) indicates that a socioeconomically disadvantaged is more sedentary during the week and a negative difference (point below the line) indicates the reverse.

For peer review only - http://bmjopen.bmj.com/site/about/guidelines.xhtml

Caption Appendix Figure A2. Inequalities in weekend sedentary time for three Scottish cohorts.

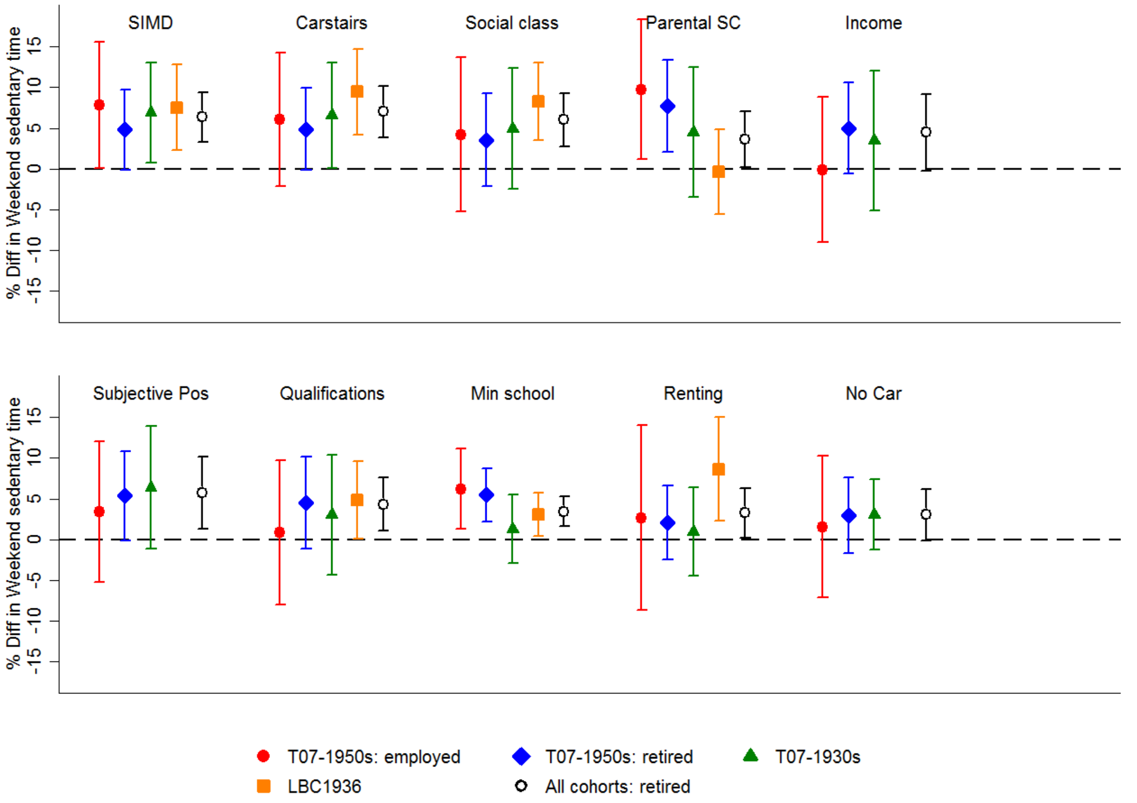

Legend: The inequalities represent SII (with 95% confidence intervals) for each of the following SEP measures (unless otherwise stated in parentheses) the Scottish Index of Multiple Deprivation, Carstairs deprivation score, Lifetime social class, Parental social class, Equivalised household income, Subjective social position, Highest educational qualification, School leaving age (- stayed on beyond minimum reference) Housing tenure (Binary measure –owners reference) and Car ownership (Binary measure – car owners reference). A positive difference (point above the dotted line) indicates that a socioeconomically disadvantaged is more sedentary during the week and a negative difference (point below the line) indicates the reverse.

APPENDIX 3: THE RELATIONSHIP BETWEEN STEP COUNT AND SOCIOECONOMIC POSITION IN OLDER ADULTS IN THREE PROPSECTIVE COHORTS

Methods

The analytical sample and methodology are the same as for the main paper with step count replacing sedentary time as the outcome. Step count was also assessed using the activPAL monitor and was transformed by taking the square root of the data to correct for positive skew. Three people were excluded from the analysis of step count as their step count may have been under recorded due to a shuffling gait.

Results

The mean step counts was as follows for the cohorts 8.9 (SD 3.7) thousand for the Twenty-07 1950s employed cohort, 8.8 (SD 3.9) thousand for the Twenty-07 1950s retired cohort, 5.1 (SD 2.8) thousand for the Twenty-07 1930s cohort and 6.9 (SD 2.8) thousand for LBC1936.

The associations between socioeconomic position and step count are shown in appendix figure 3. The results for step count tend to mirror those for sedentary time but in the opposite direction and with fewer significant relationships. This is not surprising, step count and sedentary time are fairly strongly correlated, the correlations being -0.60, -0.56, -0.51 and -0.48 for the 1950s-employed, 1950s-retired, 1930s cohort and LBC1936 respectively.

Discussion

The results for step count are similar to those for sedentary time. This is not a surprise given the moderately strong correlation. Given that we have used the activPAL data to decompose waking time into time spent sedentary, standing and walking, some correlation between step count and sedentary time is inevitable. Further analysis would benefit from taking a compositional approach modelling the components simultaneously.[8] However, that is beyond the scope of this paper.

For peer review only

Caption Appendix figure 3 Inequalities in Step Count for three Scottish cohorts.

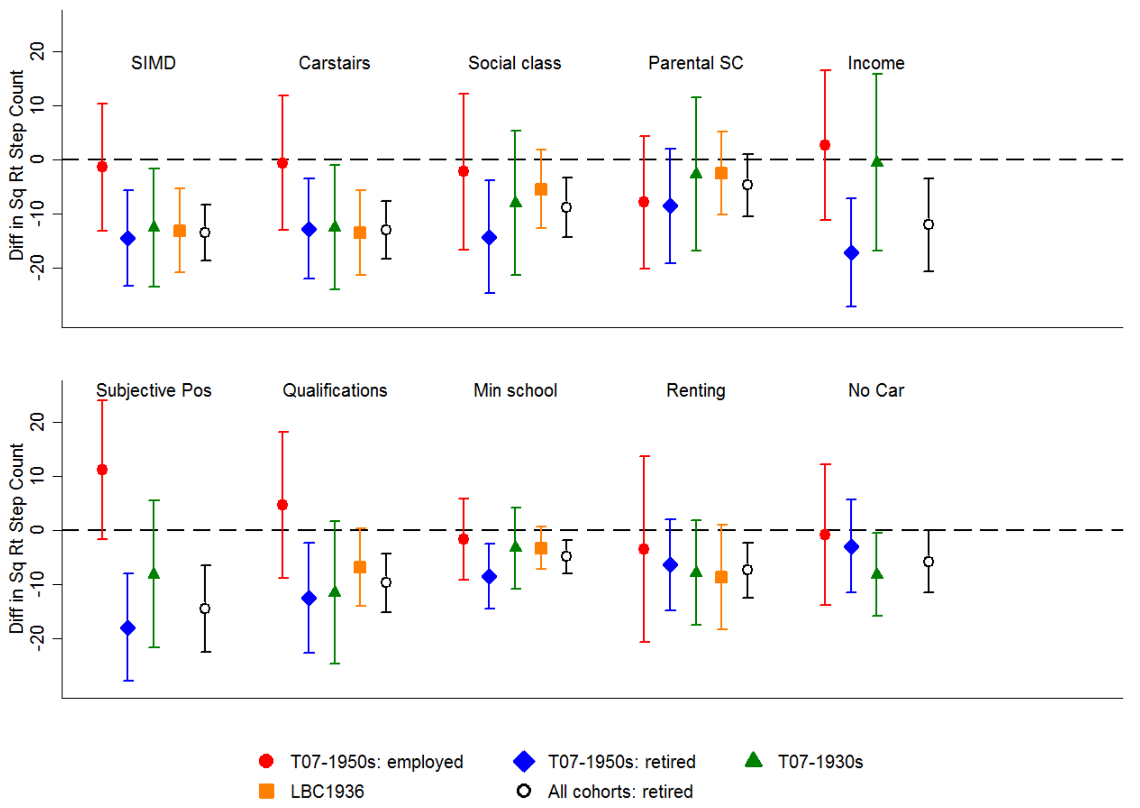

Legend: The inequalities represent SII (with 95% confidence intervals) for each of the following SEP measures (unless otherwise stated in parentheses) the Scottish Index of Multiple Deprivation, Carstairs deprivation score, Lifetime social class, Parental social class, Equivalised household income, Subjective social position, Highest educational qualification, School leaving age (- stayed on beyond minimum reference) Housing tenure (Binary measure –owners reference) and Car ownership (Binary measure – car owners reference). A negative difference (point below the dotted line) indicates that a socioeconomically disadvantaged person makes fewer steps per day a positive difference (point above the line) indicates the reverse.

For peer review only - http://bmjopen.bmj.com/site/about/guidelines.xhtml

APPENDIX REFERENCES

1 Office of Population Censuses and Surveys. London: HMSO 1980.

2 Registrar General. The Registrar General's Decennial Supplement, England and Wales 1951, Occupational Mortality Part II, Vol. 1. *Commentary HMSO, London* 1958.

3 McClements LD. Equivalence scales for children. *J Public Econ* 1977;**8**:191-210.

4 Scottish Government. Scottish Index of Multiple Deprivation. 2012.

5 Carstairs V, Morris R. Deprivation: explaining differences in mortality between Scotland and England and Wales. *BMJ* 1989;**299**:886-9.

6 Brown D, Allik M, Dundas R*, et al.* Carstairs Scores for Scottish Postcode Sectors, Datazones and Output Areas from the 2011 Census. MRC/CSO SPHSU, Glasgow. 2014.

7 Singh-Manoux A, Adler NE, Marmot MG. Subjective social status: its determinants and its association with measures of ill-health in the Whitehall II study. *Soc Sci Med* 2003;**56**:1321-33.

8 Chastin SFM, Palarea-Albaladejo J, Dontje ML*, et al.* Combined Effects of Time Spent in Physical Activity, Sedentary Behaviors and Sleep on Obesity and Cardio-Metabolic Health Markers: A Novel Compositional Data Analysis Approach. *PLOS ONE* 2015;**10**:e0139984.

For peer review only

STROBE Statement—Checklist of items that should be included in reports of ***cohort studies***

	Item No	Recommendation
**Title and abstract**	1	(*a*) Indicate the study's design with a commonly used term in the title or the abstract<br>*The term "Prospective cohort" is used in the title.*
		(*b*) Provide in the abstract an informative and balanced summary of what was done and what was found<br>*See abstract on page 2.*
**Introduction**		
Background/rationale	2	Explain the scientific background and rationale for the investigation being reported<br>*The introduction sets out rationale for the study on page 4*
Objectives	3	State specific objectives, including any prespecified hypotheses<br>*Aim of study is set out in last paragraph of introduction on page 4.*
**Methods**		
Study design	4	Present key elements of study design early in the paper<br>*The study design is described on pages 4 to 5.*
Setting	5	Describe the setting, locations, and relevant dates, including periods of recruitment, exposure, follow-up, and data collection<br>*Dates and locations are described in the first three paragraphs of the methods section on pages 4 to 5.*
Participants	6	(*a*) Give the eligibility criteria, and the sources and methods of selection of participants. Describe methods of follow-up<br>*Eligibility criteria described on page 5.*
		(*b*) For matched studies, give matching criteria and number of exposed and unexposed<br>*Not applicable.*
Variables	7	Clearly define all outcomes, exposures, predictors, potential confounders, and effect modifiers. Give diagnostic criteria, if applicable<br>*Variables are summarised on pages 5 to 6 with additional information provided in the supplementary file.*
Data sources/ measurement	8*	For each variable of interest, give sources of data and details of methods of assessment (measurement). Describe comparability of assessment methods if there is more than one group<br>*Summary of sources of data are provided in the main text file on pages 5 to 6 with more extensive details provided in supplementary document.*
Bias	9	Describe any efforts to address potential sources of bias<br>*Test for potential bias due to socioeconomic position are discussed on pages 6 and 8.*
Study size	10	Explain how the study size was arrived at<br>*This is covered on page 5 of the methods section.*
Quantitative variables	11	Explain how quantitative variables were handled in the analyses. If applicable, describe which groupings were chosen and why<br>*The variables areas described in the methods section on pages 5 to 6 and groupings discussed in the results section on page 6.*
Statistical methods	12	(*a*) Describe all statistical methods, including those used to control for confounding<br>*Statistical methods are described on page 6.*
		(*b*) Describe any methods used to examine subgroups and interactions<br>*See statistical methods section on page 6.*

For peer review only - http://bmjopen.bmj.com/site/about/guidelines.xhtml

For peer review only

		(*c*) Explain how missing data were addressed<br>*We present possible evidence of some biases on page 6 and 8.*
		(*d*) If applicable, explain how loss to follow-up was addressed<br>*We tested for potential biased on page 6 and 8.*
		(*e*) Describe any sensitivity analyses<br>*Results from alternative ways of coding independent variables are presented in supplementary file.*
**Results**		
Participants	13*	(a) Report numbers of individuals at each stage of study—eg numbers potentially eligible, examined for eligibility, confirmed eligible, included in the study, completing follow-up, and analysed<br>*This is covered on page 6 of the results .*
		(b) Give reasons for non-participation at each stage
		(c) Consider use of a flow diagram
Descriptive data	14*	(a) Give characteristics of study participants (eg demographic, clinical, social) and information on exposures and potential confounders<br>*This is presented in table 1 on page 7.*
		(b) Indicate number of participants with missing data for each variable of interest<br>*This is presented on table 1 on page 7.*
		(c) Summarise follow-up time (eg, average and total amount)<br>*Study carried out in discrete waves so not applicable.*
Outcome data	15*	Report numbers of outcome events or summary measures over time<br>*Variations in outcome are described in table 2 on page 9.*
Main results	16	(*a*) Give unadjusted estimates and, if applicable, confounder-adjusted estimates and their precision (eg, 95% confidence interval). Make clear which confounders were adjusted for and why they were included<br>*Confidence intervals are presented in the results on page 8 and in figure 1on page 16.*
		(*b*) Report category boundaries when continuous variables were categorized<br>*Not applicable.*
		(*c*) If relevant, consider translating estimates of relative risk into absolute risk for a meaningful time period<br>*Not applicable.*
Other analyses	17	Report other analyses done—eg analyses of subgroups and interactions, and sensitivity analyses<br>*Additional analyses presented as a supplementary file.*
**Discussion**		
Key results	18	Summarise key results with reference to study objectives<br>*Summary presented at start of discussion page 10.*
Limitations	19	Discuss limitations of the study, taking into account sources of potential bias or imprecision. Discuss both direction and magnitude of any potential bias<br>*Limitations discussed on page 11.*
Interpretation	20	Give a cautious overall interpretation of results considering objectives, limitations, multiplicity of analyses, results from similar studies, and other relevant evidence<br>*Discussed on pages 10 to 11.*
Generalisability	21	Discuss the generalisability (external validity) of the study results

For peer review only

For peer review only - http://bmjopen.bmj.com/site/about/guidelines.xhtml

		Generalizability is discussed on page 11.
**Other information**		
Funding	22	Give the source of funding and the role of the funders for the present study and, if applicable, for the original study on which the present article is based<br>*Funding included in the acknowledgements on page 12.*

*Give information separately for exposed and unexposed groups.

Note: An Explanation and Elaboration article discusses each checklist item and gives methodological background and published examples of transparent reporting. The STROBE checklist is best used in conjunction with this article (freely available on the Web sites of PLoS Medicine at http://www.plosmedicine.org/, Annals of Internal Medicine at http://www.annals.org/, and Epidemiology at http://www.epidem.com/). Information on the STROBE Initiative is available at http://www.strobe-statement.org.

For peer review only
